# Key Genetic Parameters for Population Management

**DOI:** 10.3389/fgene.2019.00667

**Published:** 2019-08-16

**Authors:** Robin Wellmann, Jörn Bennewitz

**Affiliations:** Animal Genetics and Breeding, Institute of Animal Science, University of Hohenheim, Stuttgart, Germany

**Keywords:** breeding values, Mendelian sampling variance, segment-based kinship, runs of homozygosity, native kinship, native contribution, native founder genome equivalent, optimum contribution selection

## Abstract

Population management has the primary task of maximizing the long-term competitiveness of a breed. Breeds compete with each other for being able to supply consumer demands at low costs and also for funds from conservation programs. The competition for consumer preference is won by breeds with high genetic gain for total merit who maintained a sufficiently high genetic diversity, whereas the competition for funds is won by breeds with high conservation value. The conservation value of a breed could be improved by increasing its contribution to the gene pool of the species. This may include the recovery of its original genetic background and the maintenance of a high genetic diversity at native haplotype segments. The primary objective of a breeding program depends on the genetic state of the population and its intended usage. In this paper, we review the key genetic parameters that are relevant for population management, compare the methods for estimating them, derive the formulas for predicting their value at a future time, and clarify their usage in various types of breeding programs that differ in their main objectives. These key parameters are kinships, native kinships, breeding values, Mendelian sampling variances, native contributions, and mutational effects. Population management currently experiences a transition from using pedigree-based estimates to marker-based estimates, which improves the accuracies of these estimates and thereby increases response to selection. In addition, improved measures of the factors that determine the competitiveness of a breed and utilize auxiliary parameters, such as Mendelian sampling variances, mutational effects, and native kinships, enable to improve further upon historic recommendations for genetic population management.

## Background

Domestic animal breeds compete with each other. Breeds with highest competitiveness increase in size and thereby displace less competitive breeds. Superior breeds are chosen by the owners because they are able to supply the demand of the consumers at low costs or achieve high-quality standards that are valued by consumers. They are thus well adapted to match consumer preferences under the current political and economic framework conditions. Selective breeding can improve the adaptation of a breed, provided that the breeding goal fulfills the requirements imposed by the consumer demands and the economic framework conditions. High genetic gain can be achieved when reliable breeding values are available for the relevant traits at an early age of the selection candidates. This requires, however, a population management strategy that achieves high selection intensities and a sufficiently short generation interval but ensures the maintenance of a high genetic diversity of the breed.

Strong selection toward an inferior breeding goal or in the absence of reliable breeding values often failed to increase the competitiveness of a breed but resulted in a substantially reduced genetic diversity. This phenomenon is known as the popular sire effect. This has happened with many companion breeds whose main breeding goal, which is to increase their physical attractiveness as pets and to improve personality traits, may be not appropriately captured by their breed standards. The reduced genetic diversity of these breeds reduced their potential to respond to selection toward new improved breeding goals and also made inbreeding unavoidable, which often led to inbreeding depression. An additional breeding goal for these breeds is thus the introduction of new genetic variation or the purging of the genetic load to get rid of inbreeding depression and to restore competitiveness.

Nowadays, many breeds are no longer able to compete with mainstream breeds. This applies to livestock species as well as companion animals. The decreasing population size causes a decline in selection intensity or a decrease in genetic diversity. The reduced genetic diversity could lead to inbreeding depression and reduces the response to selection in the long term. This further reduces their competitiveness relative to the mainstream breeds. Often, this downward spiral was set off by inappropriate or absent genetic population management or an inappropriate breeding goal. The competitiveness of small breeds is further reduced by the introduction of genomic selection, which substantially increased genetic gain only in mainstream breeds. Many small livestock breeds have been upgraded with mainstream breeds, which continued for several decades to keep them competitive and genetically diverse (Hartwig et al., 2014; Hartwig et al., 2015). This process gradually replaced their native haplotype segments, which were present in the breed before introgression, with genetic material from a few mainstream breeds. This reduced the contributions of the breeds to the genetic diversity of the species.

Rescuing endangered breeds from extinction is only possible by a conservation effort. As resources available for conservation programs are limited, breeds will compete in the future not only to match consumer preferences but also for funds from conservation programs (Boettcher et al., 2010). An optimal allocation of funds from conservation programs to specific breeds minimizes the expected conservation value of the breeds going extinct (Simianer et al., 2003). In general, the higher the value of a breed for conservation is, the more the species loses its ability to adapt to new environments after the breed has gone extinct. A parameter measuring this ability of the species is its adaptivity coverage, defined as the expected total merit index the most suitable breed has after selecting it for several generations toward a new randomly chosen breeding goal (Wellmann et al., 2014). In the future, robust and food-efficient breeds with the potential to adapt to climate change and new agro-ecological conditions and to tolerate new diseases will be needed (Kantanen et al., 2015), which requires the maintenance of a high adaptivity coverage. The optimal allocation of resources thus depends on the risks of the breeds to go extinct and on their contributions to the adaptivity coverage of the species.

The simulation study of Wellmann et al. (2014) showed that maintaining high adaptivity coverage is similar to conserving the genetic diversity of the species if many generations of selection are permissible to reach new breeding goals. The genetic diversity of a species can be decomposed into within-breed and between-breed diversity (Toro and Caballero, 2005). A long-standing debate is whether between-breed diversity is more important than within-breed diversity for prioritizing breeds for conservation (Weitzman, 1993; Meuwissen, 2009). The conservation of between-breed diversity is important because domestic breeds have, on average, an effective size of only 100 (Leroy et al., 2013), so much of the genetic diversity can be found between breeds. Within-breed diversity, on the contrary, is required for future selection response. In any case, the conservation value of a breed could be increased by prioritizing animals for breeding whose genomes are enriched with rare haplotype segments, whereby segments carrying signatures of positive selection are of particular value (Toro et al., 2009). For rare breeds that had been upgraded with mainstream breeds, this could be achieved by recovering the original genetic background of the breed, in which case the breeding program needs to ensure that enough genetic diversity remains in the breed after the introgressed genetic material has been removed (Amador et al., 2011).

In summary, population management strategies need to take different and often conflicting breeding objectives into account. The primary objective of a breeding program depends on the genetic state of the population and its intended usage. The state of a population with respect to a breeding objective can be measured by an associated genetic parameter. The relevant genetic parameters are the average breeding value for total merit, the genetic diversity of the population, and the genome equivalent that is contributed by the breed to the genetic diversity of the species. Although these parameters can be target parameters of breeding programs, their improvement can be facilitated with the help of some further auxiliary parameters. For example, the genome equivalent that is contributed by the breed to the gene pool of the species could be increased by increasing the genetic diversity at native alleles and the proportion of the gene pool that is native. Genetic gain can be increased when the Mendelian sampling variances are taken into account, which are responsible for the variances of breeding values within fulfills, and it can be further increased with the help of estimated mutational effects. Mutational effects are not only of interest to improve the accuracies of genomic breeding values but also for genome editing, which can be used to repair deleterious mutations and propagate advantageous alleles in the population (Jenko et al., 2015). The success of a breeding program depends on the accuracies with which the relevant genetic parameters have been estimated. Pedigree-based estimates are increasingly replaced by marker-based estimates, which often have substantially higher accuracies.

This review provides an overview of the key genetic parameters required for optimal population management but does not aim to give a comprehensive overview of the selection methods with which these parameters can be optimized. The paper is organized as follows. In the first section (Achieving Genetic Gain), genetic parameters are reviewed that can be taken into account to accelerate genetic progress. The considered parameters are breeding values, Mendelian sampling variances, and mutational effects. The second section (Managing Genetic Diversity) reviews parameters that are required to manage the genetic diversity of a breed and its contribution to the genetic diversity of the species. These are kinships and the founder genome equivalent (FGE) that is contributed by the breed to the gene pool of the species. The third section (Handling Introgression) reviews parameters that are important for breeds with severe genetic bottlenecks and breeds with historic introgression. These are native contributions and the kinship at native alleles. The definition of each parameter, and when appropriate a formula for predicting its value at a future time, is given. This is followed by a review of different methods for estimating the parameter and a discussion of aspects that are relevant for population management. The paper ends with a general discussion that clarifies the importance of the parameters for different types of breeding programs.

## Achieving Genetic Gain

Making the breed competitive is the most important goal of any breeding program, as it enables the maintenance of a sufficient population size for long-term survival. The competitiveness of a breed depends on its performance, health, fertility, longevity, robustness, type, behavior, and food efficiency. These trait complexes can be combined into a total merit index. The main objective of most breeding programs is to achieve a high genetic gain for total merit. This section discusses the genetic parameters that can be used for this. Although the target parameter is the average true breeding value for total merit, knowledge of individual Mendelian sampling variances and of mutational effects can help to increase selection response.

### Breeding Values

Of interest in animal breeding is not the total merit of an individual itself but the average total merit of its offspring. This is measured by its breeding value BV_*i*_, which is twice the expected deviation of the offspring performance from the population mean when mated to a large random sample of the population. It can be computed as

$$\text{B}{\text{V}}_{i}=\sum_{m}(x_{im}-2p_{m})a_{m},$$

where *a*_*m*_ is the allele substitution effect of quantitative trait nucleotide (QTN) *m* for total merit, *x*_*im*_ ∈ {0, 1, 2} is the number of copies of the alternative allele carried by individual *i* at QTN *m*, and *p*_*m*_ is the frequency of the alternative allele in a base population. A breeding program with focus on genetic gain in total merit aims to achieve a high average breeding value in the population at a future time *t* + 1. The expected genetic gain until then equals

$$\Delta G=E\left({\bar{\text{BV}}}_{t+1}\right)-{\bar{\text{BV}}}_{t}$$

where ${\bar{\text{BV}}}_{t}$ is the average breeding value of the population at time *t*. It can be computed as

$${\bar{\text{BV}}}_{t}=v_{t}^{T}BV_{t},$$

where **BV**_*t*_ is the vector with the breeding values of all individuals. Vector **v**_*t*_ contains the weights given to the individuals at time *t*. The weight *v*_*ti*_ of an individual depends on the age × sex - class *s* to which it belongs at time *t*. Thereby, the same weight is given to all individuals from the same age × sex - class. The contribution $r_{s}^{t}$ of class *s* to the population at time *t* is often assumed to be proportional to the expected number of offspring from this class that is not yet born (Meuwissen and Sonesson, 1998; Woolliams et al., 2015), so it is, in general, not proportional to the number of animals from the data set that belong to this class. The expected mean breeding value at time *t* + 1 equals

$$E\left({\bar{\text{BV}}}_{t+1}\right)={\left(r_{0}c+v_{t+1}\right)}^{T}BV_{t},$$

provided that no individual that will be born between time *t* and *t* + 1 has its own offspring in this time interval. In the above formula, *r*_0_ is the percentage of the population represented by the age cohort that will be born in this time interval, and vector **c** contains the genetic contributions of all individuals from the current population to the offspring. A primary task for a breeding program is to determine the desired frequency of use of each selection candidate *i*, i.e. the vector **c**. The result depends on the method with which the breeding values have been estimated.

**Estimates:** As most QTN and their allele substitution effects are unknown, breeding values need to be estimated from own performances, from the performances of close relatives, or from marker data.

Breeding values are traditionally obtained as the best linear unbiased predictions (BLUP) in a mixed linear model from the performances of relatives (Henderson, 1984). The model uses the additive relationship matrix **A** computed from pedigrees as the covariance matrix of the breeding values. Breeding values have this covariance structure if the randomness of the breeding values arises from the random transmission of the paternal alleles to the offspring (Fisher, 1918; Crow and Kimura, 1970). When genotypes are available, then this covariance matrix can be replaced by a covariance matrix **G** computed from genotypes. If the covariance matrix is computed by Method 1 of VanRaden (2008), then the resulting model is called the GBLUP model (Hayes et al., 2009a). This model is equivalent to a single nucleotide polymorphism (SNP) model that assumes normally distributed marker effects (Goddard, 2009). The direct genomic values obtained from a GBLUP model need to be blended with BLUP breeding values to obtain the genomically enhanced breeding values on which selection is based (VanRaden et al., 2009).

As the sources of the randomness of breeding values in different models are different, depending on whether pedigrees or genotypes are used to define the covariance matrix of the breeding values, it was a challenge to derive a model that enables the estimation of breeding values of genotyped and ungenotyped individuals in a single evaluation. One such model is single-step GBLUP (ssGBLUP), which integrates the genomic relationship matrix **G** with the additive relationship matrix **A** into a combined relationship matrix **H** (Legarra et al., 2009; Misztal et al., 2013). Fernando et al. (2014) showed that ssGBLUP is equivalent to an SNP model with normally distributed marker effects in which the genotypes of the non-genotyped individuals are imputed, and a random imputation residual is introduced to accommodate deviations between true and imputed genotypes. They also generalized the model to enable other distributions for the marker effects. Attempts have been made to further improve this SNP model by improving the imputation method and the covariance matrix of the imputation residual (Meuwissen et al., 2015).

Alternatives to GBLUP are Bayesian SNP models such as BayesA and BayesB (Meuwissen et al., 2001), BayesC (Verbyla et al., 2010), BayesCπ (Habier et al., 2011), BayesR (Erbe et al., 2012), and BayesRC (MacLeod et al., 2016), which differ in their assumptions about the distribution of marker effects. These methods are superior to GBLUP if the true distribution of marker effects deviates from the normal distribution. In this case, the model provides the highest accuracy whose prior assumptions match the genetic architecture of the trait best. Usually, MCMC algorithms are used to obtain the posterior means of the marker effects, which results in long computation times. The direct genomic values obtained from these models can then be blended with BLUP breeding values. Alternatively, genomically enhanced breeding values could be obtained directly with a Bayesian single-step method (Lee et al., 2017).

**Discussion:** While breeding programs use traditionally only genealogical information, the incorporation of genomic information enables to increase the reliability of breeding values of selection candidates at a young age. Unlike BLUP breeding values, genomic breeding values account for the Mendelian sampling term component of the breeding values of young animals, which enables selection within young full-sib families. This enables to reduce the generation interval and reduces the cost of breeding programs if progeny testing can be omitted (Schaeffer, 2006). It also enables to increase the selection intensity by large-scale genotyping and by the intense use of embryo transfer for young superior females. The latter has great potential to increase the selection intensity for some species because reliable genomic breeding values can be computed even for embryos. These factors reduce the generation interval and enable to achieve more genetic gain per generation at the same rate of inbreeding (Daetwyler et al., 2007).

As a typical quantitative trait is affected by many quantitative trait loci (QTL) with small effects (Mackay et al., 2009; Wellmann and Bennewitz, 2011a), QTL frequencies increase only slowly by artificial selection and many QTL already existed in the species at low frequencies before breed separation (Kemper et al., 2015). Most QTL are expected to show little interactions with the genetic background because sire by breed interactions are also small for most traits (Goddard et al., 2015). Phenotypes and genotypes from other breeds could therefore provide valuable information that could be used to increase the accuracies of genomic breeding values for the breed of interest.

As the LDs between QTL and non-adjacent markers in different breeds are different, high-density genotypes or even imputed whole-genome sequences are needed to take advantage of genomic data from other breeds. In this case, the number of markers is much larger than the number of QTL, so most marker effects are actually zero. As most true SNP effects are zero, a Bayesian SNP model would ideally be used that makes this prior assumption. The true distribution of an SNP effect depends on the type of control region to which the SNP belongs. Typical control regions are promoters, enhancers, insulators, and the genes themselves. Consequently, a Bayesian model for across-breed genomic prediction should be able to assume different distributions of SNP effects for different types of control region. One such model is BayesRC (MacLeod et al., 2016). This type of Bayesian model in combination with large-scale genotyping of females and across-breed genomic prediction is the recommended approach for small breeds (Hozé et al., 2014; Iheshiulor et al., 2016). Unfortunately, computation time is an issue for BayesRC and the software is currently unable to account for phenotypes of genotyped and non-genotyped individuals in a single evaluation, so the genomic breeding values need to be blended with BLUP breeding values.

As blending does not combine the different sources of information in an optimal way, a single-step evaluation may be advantageous. However, single-step evaluations usually do not take data from other breeds into account, so one has to carefully consider whether the gain in accuracy that arises from combining different information sources in an optimal way outweighs the loss of accuracy that arises from evaluating only a single breed. Whether or not data from other breeds is needed for genomic prediction depends on the population size. Large populations enable accurate predictions of genomic breeding values with GBLUP or ssGBLUP without data from other breeds if the relationships between phenotyped and genotyped individuals in the reference population and the selection candidates are high, the average relationship within the reference population is low (Pszczola et al., 2012), and the size of the reference population is large (Hayes et al., 2009b). For large breeds, the method of choice for estimating breeding values is therefore ssGBLUP. As this model is able to use geneological information and genotype information simultaneously, the accuracies of ssGBLUP and improvements thereof are usually at least as high as for any other method (Legarra et al., 2014). Exceptions are traits that are predominantly affected by few QTL with large effect (Lee et al., 2017), in which case direct genomic values are more appropriately estimated by a Bayesian SNP method or a Bayesian single-step method.

### Mendelian Sampling Variance

The breeding value BV_*k*_ of offspring *k* from sire *i* and dam *j* equals

$${\text{BV}}_{k}=\mu_{ij}+{\text{MD}}_{i}+{\text{MD}}_{j},$$

where $\mu_{ij}=\frac{{\text{BV}}_{i}+{\text{BV}}_{j}}{2}$ is his *a priori* expected breeding value, and MD_*i*_ is the random deviation from the mean that is caused by parent *i*. As maternal and paternal alleles are transmitted independently to the offspring, the variance of his breeding value BV_*k*_ can be decomposed as $\sigma_{ij}^{2}=\text{M}{\text{V}}_{i}=\text{M}{\text{V}}_{j}$, where the variance MV_*i*_ = var(MD_*i*_) of the Mendelian sampling deviation MD_*i*_ is called the Mendelian sampling variance in the offspring of individual *i*. The Mendelian sampling variance is thus responsible for the genetic variability among full sibs. Offspring of individuals that cause a high Mendelian sampling variance has less uniform breeding values, so the probability is larger that at least one of them is a top-ranking individual, which qualifies for broad use as an elite sire or dam. Consequently, individuals should be favored for breeding that have not only a high breeding value BV_*i*_ but also cause a high Mendelian sampling variance MV_*i*_.

The Mendelian sampling variance in the offspring of individual *i* equals

$${\text{MV}}_{i}=\mathrm{var}\left(\sum_{m}h_{im}a_{m}\right),$$

where **h**_***i***_ is the vector with the alleles of a randomly chosen gamete that is generated from both haplotypes of individual *i*. As alleles *h*_*im*_ and *h*_*im*__ʹ_ at adjacent QTN *m* and *m*ʹ are not transmitted independently, Mendelian sampling variances are affected by linkage. Furthermore, *h*_*im*_ is only random, when individual *i* is heterozygous at QTN *m*, so individuals with high heterozygosity cause higher Mendelian sampling variances. Thus, individual differences in the Mendelian sampling variances arise from linkage and from individual differences in the inbreeding level.

**Estimates:** The Mendelian sampling variance in the offspring of individual *i* can be estimated from his pedigree as

$${\hat{\text{MV}}}_{i}=\frac{V_{A}}{4}(1-F_{i})$$

where *V*_*A*_ is the additive variance in the base population and *F*_*i*_ is the inbreeding coefficient (Foulley and Chevalet, 1981; Dempfle, 1990). Note that the additive variance $\sigma_{At}^{2}=V_{A}(1-{\bar{F}}_{t})$ of the current population is smaller than *V*_*A*_ because the mean inbreeding ${\bar{F}}_{t}$ in the current population reduces the additive variance. This estimate of MV_*i*_ accounts for the fact that inbred individuals have lower heterozygosity, so all gametes produced by them are similar.

Genomic selection enables to obtain more accurate estimates that account not only for inbreeding but also for linkage. Estimates of Mendelian sampling variances can be obtained by simulating gametes (Segelke et al., 2014) or by a prediction formula such as

$${\hat{\text{MV}}}_{i}=a^{T}\Omega_{\text{i}}a,$$

where **Ω**_**i**_ = var(**h**_***i***_) is the covariance matrix of the genotypes of the gametes that are produced by individual *i*, and **a** is the vector with allele substitution effects (Bonk et al., 2016).

**Discussion:** To increase response to selection and the probability of breeding a top-ranking individual, breeders aim to arrange matings that produce offspring with high breeding values. The breeding value of the offspring should surpass the average breeding value ${\bar{\text{BV}}}_{t+g}^{s}$ of his competitors at the time *t* + *g* when he could be used for breeding. The competitors of the offspring are the future breeding individuals of the same sex *s*. The optimal way to account for Mendelian sampling variances in mate allocation depends therefore on the desired sex *s* of the offspring. As sexed semen is available for many species, matings with female offspring and matings with male offspring can indeed be planned independent from each other.

The estimate ${\bar{\text{BV}}}_{t+g}^{s}$ can be obtained by extrapolating the historic development of the average breeding values of breeding males or females in the future, whereby the time *g* that passes until the offspring could be used for breeding depends on his sex and equals approximately the generation interval.

According to this breeding strategy, matings would be arranged such that sire *i* is chosen for dam *j* if the breeding value BV_*k*_ of his offspring *k* surpasses the threshold value ${\bar{\text{BV}}}_{t+g}^{s}$ with the highest probability.

The goal is thus to arrange matings for which the offspring *k* maximizes

$$P(\text{B}{\text{V}}_{k}>{\bar{\text{BV}}}_{t+g}^{s})=1-F\left(\frac{{\bar{\text{BV}}}_{t+g}^{s}-\mu_{ij}}{\sqrt{\sigma_{ij}^{2}}}\right),$$

where *F* is the standardized cumulative distribution function. The above formula shows that the probability to produce a superior offspring is monotonically increasing in

(1) $$I_{ij}=\frac{\mu_{ij}-{\bar{\text{BV}}}_{t+g}^{s}}{\sqrt{\sigma_{ij}^{2}}},$$

so the above approach is equivalent to selecting the male *i* for dam *j* that maximizes *I*_*ij*_.

To make selection decisions for males and females independent from each other, the values *μ*_*ij*_ and $\sigma_{ij}^{2}$ in the above formula can be replaced by their average values, whereby the averages are taken over all individuals from the opposite sex. If selection candidate *i* is a male, then the average values are $\mu_{io}=\frac{{\text{BV}}_{i}+{\bar{\text{BV}}}_{t}^{f}}{2}$ and $\sigma_{io}^{2}={\text{MV}}_{i}+\frac{\sigma_{At}^{2}}{4}$, respectively, where ${\bar{\text{BV}}}_{t}^{f}$ is the current average breeding value of potential dams. This provides the index

$$I_{io}=\frac{\mu_{io}-{\bar{\text{BV}}}_{t+g}^{s}}{\sqrt{\sigma_{io}^{2}}},$$

on which the selection of breeding males could be based. The formula shows that a high Mendelian sampling variance is advantageous if the expected breeding value of the offspring *μ*_*io*_ is smaller than the threshold value ${\bar{\text{BV}}}_{t+g}^{s}$, but it is disadvantageous if *μ*_*io*_ is larger than the threshold value. The reason is as follows. If the expected breeding value *μ*_*io*_ is higher than the threshold, then the realized breeding value BV_*k*_ is more likely also above the threshold if it deviates little from the expected value. In contrast, if the expected breeding value *μ*_*io*_ is lower than the threshold, then the realized breeding value BV_*k*_ is more likely above the threshold if it deviates much from the expected value.

An index may be advantageous that gives the same weight to the Mendelian sampling variance, no matter what the expected breeding value *μ*_*io*_ of the offspring is. Such an index can be obtained by approximating *I*_*io*_ with a function that is linear in *μ*_*io*_ and *σ*_*io*_. As shown in the **Supplementary Material**, the linear approximation of index *I*_*io*_ in the vicinity of the average values for *μ*_*io*_ and σ_*io*_ is a monotone function of

(2) $$I_{i}=\text{B}{\text{V}}_{i}+\lambda_{s}\sqrt{2{\text{MV}}_{i}+\frac{\sigma_{At}^{2}}{2}},$$

where the weight *λ*_*s*_ depends on the desired sex *s* of the offspring. It can be computed as

$$\lambda_{s}=\frac{2\Delta G_{s}\pm \Delta S_{s}}{\sigma_{At}},$$

where ∆*G*_*s*_ is the expected genetic gain for total merit in the next generation (i.e. within *g* years) and ∆*S*_*s*_ is the difference between the average breeding values of sires and dams that are eligible as parents of individuals with sex *s*. The value ∆*S*_*s*_ is added when a male offspring is desired and subtracted for female offspring. As the selection intensity for males is typically higher than the selection intensity for females, ∆*S*_*s*_ is positive. Consequently, accounting for the Mendelian sampling variance is more important for producing superior male offspring than for producing superior female offspring.

It can be seen by simulating values for BV_*i*_ and MV_*i*_ that the variance of the first term in Equation 2 is much larger than the variance of the second term. This shows that the Mendelian sampling variance is of minor importance in a breeding program. However, in a population under selection, the variance of BV_*i*_ is reduced by the Bulmer effect (Bulmer, 1971), and it is further reduced because only individuals are taken into consideration that seem eligible as parents because of their high breeding values. The Mendelian sampling variance is not affected by the Bulmer effect (Van Grevenhof et al., 2012), so the high ∆*G*_*s*_ in populations under strong selection causes the variance of the second term to increase. All these factors increase the relative importance of the Mendelian sampling variance for populations under strong selection, especially for breeders that aim to breed top-ranking individuals. It can thus be recommended to base selection decisions for breeding males on the index *I*_*i*__o_ or on the index *I*_*i.*_

Bijma et al. (2018) proposed to select individuals for breeding based on the alternative but similar index

$${\tilde{I}}_{i}={\text{BV}}_{i}+x_{p}\sqrt{2\text{M}{\text{V}}_{i},}$$

which showed good performance in a simulation study. Here, *x*_*p*_ is the standardized truncation point belonging to the selected proportion *p*.

### Mutational Effects

The mutational effect *a*_*m*_ of QTN *m* is the average effect on the genotypic value of substituting a “wild-type” allele that is randomly chosen from the population by the mutant allele. Knowledge of mutational effects is useful not only to improve estimates of genomic breeding values and Mendelian sampling variances but also for genome management. The aim of genome management is to repair deleterious mutations and introduce advantageous mutations into the population by genome editing.

**Estimates:** As livestock breeds have been selected for a long time, most QTNs that affect the selected traits have already been fixed, are pleiotropic, or have small effects. Hence, the QTNs segregating in the population predominantly have small effects that are difficult to detect. Establishing a pipeline for the discovery of causal QTN and for estimating their effects is an active area of research (Hickey et al., 2016).

Mutational effects can be estimated from multi-breed sequence data with a Bayesian method such as BayesRC (MacLeod et al., 2016). However, as QTN effects are typically small, and the effective sizes of single breeds are low, the accuracy of the estimates and the mapping precision may not be sufficient. The accuracy and mapping precision can be increased by accounting for prior knowledge about the probability that particular mutations are deleterious or advantageous. Most novel mutations are neutral or deleterious (Eyre-Walker and Keightley, 2007). The probability that a particular mutation is deleterious can be estimated from the conservation scores of the SNPs, from the type of control region an SNP belongs to as indicated, for example, by histone modifications (Huang et al., 2017), from the age of the mutation and the probability for deleterious mutations to have been purged (Leberg and Firmin, 2008), and from a lack of homozygous individuals (Pausch et al., 2015; Derks et al., 2017).

Further methods for the estimation of QTN effects include testing mutations in a cell line with massively parallel reporter assays (Melnikov et al., 2012) and the prediction of mutational effects with deep neural networks (Paggi et al., 2017). This also enables the discovery of novel mutations that would increase total merit even more than the SNPs that are segregating in the population. A further method to improve upon the segregating mutations is phage-assisted continuous evolution (Lane and Seelig, 2014).

**Discussion:** Knowledge about mutational effects can be used for the estimation of genomic breeding values for genetic load and for conventional traits, but it can also be used to repair deleterious mutations and propagate advantageous mutations by genome editing. The development of low-cost multiplexed edits in zygotes makes genome editing affordable (Qin et al., 2015). Changing specific nucleotides in the genomes of germ-line cells by genome editing has shown to have great potential to increase genetic gain in livestock. For example, turning 20 QTN for the top 10 sires into the advantageous alleles per generation could almost double genetic gain in a simulation study (Jenko et al., 2015). This requires, however, that the causal QTN can be identified (Simianer et al., 2018). The methods mentioned above may not only be able to find the causal mutations but also may even improve upon the mutations segregating in the population, in which case genetic gain would increase even more than predicted (Jenko et al., 2015). However, editings that had been predicted to be advantageous may in fact be deleterious in some cases. Therefore, testing them in the heterozygous and homozygous states in living animals, and undoing edits that turned out to be deleterious, needs to be part of this breeding strategy. Further research is needed to improve upon the existing methods for QTN detection.

## Managing Genetic Diversity

Domestic species need to adapt to the requirements that arise from changes in political and economic framework conditions, climate change, changes in consumer preferences, and disease dissemination. Genetic diversity enables a species to adapt to these changes. The genetic diversity of a domestic species depends on the diversity of the gene pool of each breed and on the overlap between the gene pools of different breeds. Of interest for population management are therefore the genetic diversity of the target breed and a measure for the part of its gene pool that does not overlap with other breeds. This non-overlapping part is a reservoir for advantageous mutations that could be detected and brought into other breeds by genome editing or conventional breeding methods. It can be measured by the FGE that is contributed by the target breed to the gene pool of the species. A high contribution ensures genetic uniqueness of the breed and keeps it a valuable resource for the detection of rare advantageous alleles.

The genetic diversity of many breeds is threatened because high genetic gain was realized by a too high selection intensity. In a population with high selection intensity, only a few individuals are used as parents of the next generation, which results in a low effective population size *N*_*e*_. The sampling of individuals for breeding and the random transmission of alleles from parents to the offspring cause random allele frequency changes from one generation to the next. This genetic drift leads to the loss of segregating alleles and thus to the loss of genetic diversity. The realization of genetic gain and the maintenance of high genetic diversity are therefore conflicting breeding goals.

The loss of genetic diversity that is observed in many breeds not only reduces their potential to adapt to changes in their environments and reduces selection response in the long term but also causes the homozygosity of the individuals to increase. The increased homozygosity increases the probability that recessive deleterious alleles are homozygous, in which case they have an effect on the phenotype. Deleterious mutations outnumber advantageous mutations and loss-of-function alleles are usually recessive, so the overall effect of an increased homozygosity on the phenotype is deleterious. The increased homozygosity causes the fitness and fertility of the breed to decrease, which may also cause a decrease in performance. The decrease in fitness, fertility, and performance that is associated with an increased level of inbreeding is called inbreeding depression.

Consequently, breeding programs need to control the genetic diversity of the breed to enable response to selection in the long term and to enable breeders to avoid inbreeding depression. Genetic diversity can be controlled by selecting individuals with rare haplotype segments for breeding, which are the individuals that have a small average kinship with the population. The genetic diversity of a population can therefore be managed by maintaining a small average kinship between the individuals. The target parameters for population management that enable to control genetic diversity are therefore the kinships between individuals and the FGE that is contributed by the breed to the gene pool of the species. The notions of kinship and FGE both rely on the concept of identical by descent (IBD).

### Concept of IBD

The concept of IBD was established by Malécot (1948) and applied to pedigree data, which is a special case of the general concept described in this section. Two alleles from the same locus are said to be IBD if they descend from the same haplotype of a common ancestor who fulfills specific criteria. Different notions of IBD arise depending on the criterion that needs to be fulfilled by the common ancestor. The imposed criterion could be

- The common ancestor belongs to a predefined set of individuals, or
- The ancestral allele and the alleles of interest belong to identical haplotype segments, or
- The allele did not mutate in the genetic lineages from the ancestor to the descendants.

Genetic parameters that rely on the concept of IBD refer to a base population. The base population is assumed to be placed so far back in time that any population structure in the base population is negligible for the current population. That is, all founders, i.e. all individuals from the base population, can assumed to be equally related. The base population has the property that individuals that lived earlier contribute little to the estimates. Hence, a common ancestor of two individuals from the current population can be considered older than the base population if an allele that was transmitted from the ancestor to both individuals *via* separate paths is not likely to satisfy the criterion for being IBD in the descendants. This approach enables to define the age of the base population for all notions of IBD. As all individuals from the base population are assumed to be equally related, the allele frequencies in the base population are the actual allele frequencies of a population in which all founder alleles have an equal probability for being IBD. The different criteria that could be imposed for IBD alleles are discussed in detail below.

**Criterion (i):** When pedigrees are used to estimate genetic parameters, then a base population is defined as the set of all founders, which are the individuals with unknown parents. As the alleles of the founders are assumed to be pairwise different, two alleles are IBD if they originate from the same founder allele. The base population is in this case only several decades in the past.**Criterion (ii):** When marker data is used for estimating genetic parameters, then the concept of IBD is usually based on haplotype segments. Two alleles of an individual belong to the same haplotype if they are inherited from the same parent. Thus, the genome *H*_*i*_ = {**m**_***i***_, **p**_***i***_} of each individual *i* consists of two haplotypes, a maternal haplotype **m**_***i***_ and a paternal haplotype **p**_***i***_. The identification of these haplotypes requires to phase the marker data. The alleles at each position are assumed to be bi-allelic and could be coded as 0 or 1 depending on whether the wild-type allele or the alternative allele is present. In this context, two alleles from the same locus chosen from two haplotypes are **IBD** if they are contained in identical haplotype segments. The segments in which two haplotypes coincide are called **IBD** segments or runs of homozygosity (ROH; Peripolli et al., 2016). For two haplotypes **h**_1_, and **h**_2_, we denote the set of markers that are contained in IBD segments as IBD(**h**_1_, **h**_2_).

Segments in which two haplotypes coincide are inherited from a common ancestor, whereby long haplotype segments predominantly originate from recent common ancestors. In general, *g* generations after the base population has been established, the length of single-path IBD segments is approximately exponentially distributed with a mean of $\frac{100}{2g}$ cM (Browning, 2008). Thus, when only IBD segments with a length of at least *m* cM are taken into account, the base population can assumed to be about $\frac{100}{2m}$ generations in the past. However, as the length of IBD segments is random, some IBD segments are even older, whereas others are younger, but are already too short for being captured.

Haplotype segments are usually determined from marker data, which cover the whole genome but do not include all base pairs. Therefore, IBD segments are required to contain a minimum number of markers to ensure that the marker alleles do not coincide by chance. The number of markers required to ensure that two segments with identical marker alleles are IBD depends on the distribution of allele frequencies and is often considered to be 20 (Peripolli et al., 2016).

If the base population should be located *g* generations in the past, then segments of length $\frac{100}{2g}\text{ cM}$ need to be detectable. As segments are required to contain at least 20 markers, this is ensured if the minimum distance between adjacent markers is $\frac{100}{40g}\text{ cM}$. For example, the genome size in cattle is 32.5 Morgan with 1.25 cM/Mb (Arias et al., 2009). If the base population is located *g* = 20 generations in the past, then segments of length $\frac{100}{2g}=2.5\text{ cM}$ need to be detectable, which equals approximately 2.0 Mb. This requires a marker distance of $\frac{100}{40g}=0.125\text{ cM}$. If the markers from the marker panel are exactly equally spaced, then this requires at least a $\frac{32.5}{0.01\cdot 0.125}=26\text{ K}$ marker panel. As the markers in the bovine marker panels are not exactly equally spaced, a 50K panel would be appropriate. This is in accordance with the study of Ferenčaković et al. (2013), who showed that the accuracy of detection is insufficient with the 50K marker panel for segments shorter than 2 Mb but high for segments longer than 4 MB.

**Criterion (iii):** If the criterion for IBD is that no mutation has occurred, then IBD alleles are also said to be identical by state (IBS). It can easily be calculated from the low mutation rate in mammals that the base population is located in this case several million years in the past. As all other criteria of IBD assume a much more recent base population, IBS alleles are not necessarily IBD with respect to other notions of IBD. According to Gómez-Romano et al. (2013), a minimum marker density of 3*N*_*e*_ SNP/Morgan is required to get IBS estimates that are sufficiently accurate for practical purposes.

### Kinships

An important aspect of any animal breeding program is the avoidance of inbreeding depression. Inbreeding depression is, on average, proportional to the expected inbreeding coefficient of the individual, which is equal to the kinship of its parents. The kinship of two individuals *i*, *j* is defined as the probability that two alleles *X*_*i*_*,Y*_*j*_, randomly chosen from the same locus from both individuals, are IBD (Malécot, 1948; Caballero and Toro, 2000). That is,

$$f_{\text{IBD}}(i,j)=P(X_{i}\overset{\text{IBD}}{=}Y_{j})$$

Keeping the mean kinship ${\bar{f}}_{{\text{IBD}}_{t}}$ of the population low is often achieved by restricting its rate of increase per generation ∆*f*_*g*_. For populations that have undergone serious genetic bottlenecks, an alternative approach aims to increase the genetic diversity ${\text{Div}}_{t}=1-{\bar{f}}_{{\text{IBD}}_{t}}$ of the population, whereby the expected genetic gain for the genetic diversity between time *t* and *t* + 1

$$\Delta {\text{Div}}_{t}={\bar{f}}_{{\text{IBD}}_{t}}-E({\bar{f}}_{{\text{IBD}}_{t+1}})$$

is negative for most populations because rare alleles get lost due to random genetic drift. However, the effect of historic genetic drift could partly be reversed in a breeding program or allele frequencies could be equalized, which both causes the genetic diversity to increase. The current mean kinship of the population is

(3) $${\bar{f}}_{{\text{IBD}}_{t}}=v_{t}^{T}f_{t}v_{t},$$

where **f**_*t*_ is the matrix with pairwise kinships of the individuals from the population. The expected mean kinship of the population at time *t* + 1 is

(4) $$E({\bar{f}}_{{\text{IBD}}_{t+1}})={\left(r_{0}c+v_{t+1}\right)}^{T}f_{t}(r_{0}c+v_{t+1})+l_{\text{IBD}}(c),$$

where *l*_IBD_(**c**) is the linear correction term defined in the **Supplementary Material**. The purpose of the correction term can easily be seen by inspecting the following formula for the expected mean kinship in a population with non- overlapping generations:

$$E\left({\bar{f}}_{{\text{IBD}}_{t+1}}\right)=c^{T}f_{t}c+\frac{1-c^{T}d(f_{t})}{2N}$$

Here, vector *d*(**f**_***t***_) contains the self-kinships, which are the diagonal elements of matrix **f**_*t*_, and *N* is the population size (Wellmann and Pfeiffer, 2009). The summand **c**^*T*^**f**_*t*_**c** accounts for the redistribution of allele frequencies in accordance with the contributions of the selection candidates. The right summand causes an additional increase of the mean kinship, which arises from the random Mendelian sampling of the alleles that are passed to the offspring. This additional increase results from allele frequency changes due to random genetic drift. The right term vanishes for highly inbred parents because individuals that carry the same alleles at both haplotypes pass always the same alleles to their offspring. Hence, minimizing the mean kinship in the population favors the use of inbred individuals for breeding. The same has been observed for breeding programs that aim to maximize the variance of the genotypic values (Cervantes and Meuwissen, 2011). This is, however, not a desirable feature of a breeding program because inbred individuals tend to suffer from inbreeding depression and a high Mendelian sampling variance is considered desirable for breeding programs that select for total merit. Therefore, instead of using the above formula, the summand on the right-hand side is usually neglected for making selection decisions, e.g. by replacing the vector **c** in the right summand by a vector with uniform contributions.

**Estimates:** The pedigree-based kinship estimate *f*_PED_(*i*, *j*) of individuals *i*, *j*, which is also called their genealogical coancestry, was used in animal breeding since Cotterman (1940) and Malécot (1948) generalized Wright’s coefficient of inbreeding. The kinship is defined with respect to a base population in which all individuals are assumed to be unrelated and non-inbred. The individuals from the base population are called founders, so *f*_PED_(*k*, *k*) = 0.5 and *f*_PED_(*k*, *l*) = 0 for all founders *k*, *l*. The kinship estimates of descendants *i*, *j* are obtained as

$$\begin{array}{c} f_{\text{PED}}(i,\;i)=\frac{1}{2}(1+f_{\text{PED}}(s_{i},\;d_{i})),\\ f_{\text{PED}}(i,j)=\frac{1}{2}(f_{\text{PED}}(s_{i},j)+f_{\text{PED}}(d_{i},j)), \end{array}$$

where *s*_*i*_ is the sire and *d*_*i*_ is the dam of individual *i* (Boyce, 1983). Although the pedigree-based kinship is the expected proportion of IBD alleles, the true proportion deviates from this value due to the random transmission of alleles in the genetic lineages from the founders to the individuals of interest. This results in an estimation error that can be avoided when kinships are estimated from marker data.

Nejati-Javaremi et al. (1997) defined a marker-based estimate by applying the definition of Malécot (1948) to genetic markers. This estimate, which is now called the molecular kinship *f*_MOL_(*i*, *j*) between individuals *i* and *j*, is the probability that two marker alleles, taken at random from both individuals, are equal. The molecular kinship has the disadvantages that the selection of subpopulations for SNP discovery and the preselection of markers for inclusion in the marker panel could cause an ascertainment bias and that the estimates are not directly comparable to pedigree-based estimates because the ages of the base populations differ. Although the ascertainment bias can be diminished by LD-based pruning (Malomane et al., 2018), this kinship estimate has further important disadvantages, which are discussed below.

Another parameter frequently used as a kinship estimate is the covariance of genomic breeding values. According to Method 1 of VanRaden (2008), the covariance of the genomic breeding values of individuals *i* and *j* is

$$G_{ij}=\frac{1}{\Sigma_{m}2p_{m}(1-p_{m})}\;\sum_{m}(x_{im}-2p_{m})(x_{jm}-2p_{m}),$$

where *x*_*im*_ ∈ {0, 1, 2} is the number of copies of the alternative allele carried by individual *i* at SNP *m*, and *p*_*m*_ is the frequency of the alternative allele in the base population. The covariance *G*_*ij*_ is a popular kinship estimate because it is easy to compute when the allele frequencies in the base population are known. Moreover, $E\left(\frac{1}{2}G_{ij}\right)$ equals the pedigree-based kinship when the genotypes are considered random, and the base population is placed far enough back in time so that any population structure of the base population can be neglected (Habier et al., 2007).

The most useful marker-based kinship estimate seems to be the segment-based kinship between individuals *i* and *j*, which equals the probability that two alleles, taken at random from both individuals from the same locus, belong to identical haplotype segments. We assign a genomic window of length *L*_*m*_ to each marker *m* from the marker set ℳ and define the segment-based kinship dependent on a subset M⊂ℳ of markers, which represents a particular part of the genome. This enables to compute different kinship estimates for different parts of the genome. This approach was popularized by Gómez-Romano et al. (2016) as it enables to control the inbreeding level differently at different genome parts.

The set IBD(**h**_1_, **h**_2_) consists of all markers from shared segments of haplotypes **h**_1_ and **h**_2_, so the proportion of genome part *M* for which the two haplotypes are IBD is

$$f_{\text{SEG}}(h_{1},\;h_{2};M)=\frac{\sum_{m\in M\cap \text{IBD}(h_{1},\;h_{2})}L_{m}}{\sum_{m\in M}\;L_{m}}.$$

This value is called the segment-based kinship between haplotypes **h**_1_ and **h**_2_ at genome part *M*. The average segment-based kinship between two haplotype sets ${ℋ}_{1}$ and ${ℋ}_{2}$ at genome part *M* is

$$\bar{f_{\text{SEG}}}({ℋ}_{1},\;{ℋ}_{2};M)=\frac{1}{\left|{ℋ}_{1}|\;|{ℋ}_{2}\right|}\sum_{h_{1}\in {ℋ}_{1}}\sum_{h_{2}\in {ℋ}_{2}}f_{\text{SEG}}(h_{1},\;h_{2};M),$$

where $\left|{ℋ}_{1}\right|$ is the number of haplotypes in set ${ℋ}_{1}$. The segment-based kinship *f*_*SEG*_(*i*, *j*) between two individuals *i* and *j* is the average kinship between their haplotype sets *H*_*i*_ and *H*_*j*_, so

$$f_{\text{SEG}}(i,j)=\bar{f_{\text{SEG}}}(H_{i},\;H_{j};ℳ).$$

This definition coincides with the definition of de Cara et al. (2013a). The segment-based kinship is thus the expected proportion of cases for which two randomly chosen alleles, one from each individual, belong to identical segments. The ROH-based inbreeding coefficient of individual *i*

$$F_{\text{SEG}}(i)=f_{SEG}(p_{i},\;m_{i};\;ℳ)$$

is the proportion of its genome for which the paternal haplotype **p**_*i*_ and the maternal haplotype **m**_*i*_ are IBD. Calculating the segment-based kinship requires phased genotypes, which can be obtained, for example, with Beagle (Browning and Browning, 2007). In contrast, calculating the ROH-based inbreeding coefficients does not require phased genotypes. As shown in the previous section, estimates of genetic parameters refer to a base population if the notion of IBD is based on shared segments, which is the case for the segment-based kinship. The age of the base population depends on the minimum length of IBD segments. The length needs to be sufficiently low such that any population structure in the base population can be neglected. This is of particular importance for multi-breed evaluations, in which case the base population needs to be placed before breed formation. This requires to capture short IBD segments for which high-density marker data are needed. As the individuals in a base population are assumed to be unrelated, minimizing the segment-based kinship aims to re-establish the allele frequencies of the base population.

Minimizing the segment-based kinship or the covariance of genomic breeding values aims to reverse random genetic drift. This means in the absence of selection to re-establish the allele frequencies from the base population, which also drives the frequencies of recessive deleterious alleles toward their historic low values and thus increases fitness. In contrast, minimizing the molecular kinship aims to drive the alleles toward intermediate frequencies, which also drives the frequencies of recessive deleterious alleles toward intermediate values. This increases the probability that recessive deleterious alleles are homozygous and thus reduces fitness (de Cara et al., 2013a). Managing the molecular kinship of a population cannot therefore be recommended, except for breeding programs that simultaneously purge genetic load. In principle, the segment-based kinship and the covariance of genomic breeding values could both be used to re-establish the allele frequencies of the base population. However, these allele frequencies are usually unknown, which is a problem because they are required to compute the covariance *G*_*ij*_ of the genomic breeding values. Using the actual allele frequencies instead is not an alternative because the historic allele frequencies would not be re-established in this case. This makes the covariance of genomic breeding values unsuitable as a kinship estimate, which is also confirmed by Henryon et al. (2018). Additional reasons are that the preselection of markers for inclusion in the marker panel could cause an ascertainment bias and that the covariances may not estimate the true kinship with sufficient accuracy. Indeed, the correlations between pedigree-based inbreeding coefficients and inbreeding coefficients obtained from genomic covariances are usually lower than their correlations with the ROH-based inbreeding coefficients. When the genomic covariance matrix is defined by Method 2 of VanRaden, then the correlations are sometimes close to zero (Zhang et al., 2015). The recommended kinship estimate for genotyped individuals is therefore the segment-based kinship.

The kinship estimates can be used to compute the mean kinship of the current population with Formula 3 or with the alternative formula given in the **Supplementary Material**, which accounts for different sources of bias. Formula 4 overestimates the average kinship of the population at time *t* + 1 slightly if segment-based estimates are inserted. This is because recombination breaks haplotype segments into smaller pieces until they are so short that they no longer contribute to the kinship estimates. This problem could be circumvented by reducing the minimum permissible length of a segment annually, so that the time at which the base population lived remains constant. Moreover, in a population under strong selection, the increase in kinship may be larger than predicted due to the directed changes in allele frequencies resulting from selection (De Beukelaer et al., 2017).

**Discussion:** As the expected inbreeding coefficient of offspring *k* equals the kinship between its sire *s*_*k*_ and dam *d*_*k,*_ i.e.

$$E(F_{\text{IBD}}(k))=f_{\text{IBD}}(s_{k},\;d_{k}),$$

an important decision for any breeding program is whether related or unrelated individuals should be mated.

The rationales between mating related individuals are first that the increased homozygosity of the offspring exposes recessive deleterious alleles to selection, which enables to exclude carriers of these alleles from breeding and thus purges genetic load. Second, the offspring are more uniform than the offspring of unrelated parents, which makes their genotypes more predictable and thus enables breeders to establish uniform herds. Third, this approach enables breeders to use non-additive genetic variance. Fourth, mating of related parents eventually causes the population to split into several subpopulations, which eventually leads to the fixation of different alleles in the different subpopulations. This approach in combination with an equalization of family sizes is therefore the most effective way to conserve genetic diversity, provided that the subpopulations do not go extinct because of reduced fitness (Kimura and Crow., 1963; Cervantes et al., 2016). In most breeds, however, inbreeding depression is due to the cumulative effect of many deleterious alleles, which makes purging of genetic load unfeasible. Exposing carriers of deleterious alleles to selection by mating related individuals cannot therefore be recommended as a breeding strategy (Boakes et al., 2007; de Cara et al., 2013b).

An alternative approach is to facilitate the mating of unrelated individuals. The rationales behind this approach are first that the increased heterozygosity causes the offspring to suffer less from inbreeding depression. Second, the offspring would have progeny with less uniform breeding values, which could make him more suitable as a parent of top-ranking individuals. Third, this approach keeps the population unstructured, which also keeps the number of potential mating partners for each individual high. This is therefore the recommended approach for breeding programs that aim to achieve high genetic gain.

The mating of related individuals can only be avoided if the mean kinship of the population is low. This is not the case for breeds that have undergone serious genetic bottlenecks, so the mean kinship of such breeds needs to be reduced, which can be achieved by minimizing the average segment-based kinship of the population and by genetic introgression. For all other breeds, it may be sufficient to restrict the rate of increase of the mean kinship. The rate of increase in mean kinship per generation ∆*f*_*g*_ determines the effective population size *N*_*e*_ *via* the formula $\Delta f_{g}=\frac{1}{2N_{e}}$. It is therefore common to restrict the increase in mean kinship in accordance with the desired effective size *N*_*e*_. In the literature, there seems to be a consensus that a population with an effective size between 50 and 100 is long-term viable (Meuwissen, 2009). Usually, *N*_*e*_ = 100 is recommended to be on the safe side. A population maintains an effective size of at least *N*_*e*_ if the rate of increase in mean kinship per generation ∆*f*_*g*_ is at most $\frac{1}{2N_{e}}$. Hence, for a population with generation interval *L*, the kinship of the population at time *t* + 1 should satisfy

$$E\left({\bar{f}}_{{\text{IBD}}_{t+1}}\right)\leq 1-\left(1-{\bar{f}}_{IBD_{t^{\prime }}}\right){\left(1-\frac{1}{2N_{e}}\right)}^{\frac{t-t^{\prime }+1}{L}}$$

(Woolliams et al., 2015), where *t*ʹ is the time at which the breeding program started.

### Founder Genome Equivalent

The genetic diversity of the species can be approximated by the genetic diversity of a hypothetical multi-breed population, which would ideally include all existing breeds. This multi-breed population is called the core set. The contributions of the breeds to the core set are chosen such that they simultaneously maximize its genetic diversity and minimize its average kinship (Eding and Meuwissen, 2001; Eding et al., 2002). The average kinship of the core set at time *t* is

$$f_{{\text{CORE}}_{t}}=b_{t}^{T}f_{t}b_{t},$$

where matrix **f**_*t*_ contains average kinships within and between breeds, and vector **b**_*t*_ contains the breed proportions. Increasing the genetic diversity of the core set is equivalent to increasing its FGE, which is the minimum size of a gene pool that has the same genetic diversity as the core set. Such a gene pool would consist of unrelated genomes. As individuals with unrelated genomes are commonly called founders, this parameter is called the FGE of the core set. It can be computed as

$${\text{FGE}}_{t}=\frac{1}{2f_{{\text{CORE}}_{t}}}.$$

An important genetic parameter for a conservation program is the FGE that is contributed by the target breed to the gene pool of the core set, which is

$${\text{conFGE}}_{t}=\text{FG}{\text{E}}_{\text{t}}-{\text{FGE}}_{t}^{*}.$$

Thereby, ${\text{FGE}}_{t}^{*}$ is the FGE of a multi-breed core set that does not include the target breed. The expected increase of the FGE that is contributed by the target breed to the core set between times *t* and *t* + 1 equals

$$\Delta {\text{conFGE}}_{t}\approx E\left({\text{FGE}}_{t\;+\;1}\right)–{\text{FGE}}_{t},$$

whereby equality holds if only the genomes of the target breed are managed.

**Estimates:** Estimating the FGE of the core set requires kinship estimates, which have been defined in a previous section. The kinship estimates need to refer to a base population that is placed before breed formation such that any population structure in the base population is negligible. This enables the kinship to capture relationships between different breeds that arose from common ancestors that lived before breed formation. Pedigree-based estimates are unsuitable because pedigree recording started after breed formation. Segment-based estimates are suitable if they are calculated from a high-density marker panel. As shown in a previous section, the minimum length of a segment can then be chosen such that the base population has the desired age. The molecular kinship computed from whole-genome sequences refers to a base population that could be located millions of years in the past. To facilitate an interpretation of results obtained with this kinship, the estimates would need to be transformed such that they refer to a base population of appropriate age.

**Discussion:** A high increase in ∆conFGE_t_ can be achieved by minimizing the expected kinship $E\left(f_{{\text{CORE}}_{t+1}}\right)$ of the core set at time *t* + 1. If only the target breed is managed, then this optimization problem is equivalent to minimizing the objective function

$$b_{k}E({\bar{f}}_{{\text{SEG}}_{t+1}})+(1-b_{k})2E({\bar{f}}_{{\text{REL}}_{t+1}}),$$

where the contribution *b*_*k*_ of the target breed is a constant, and ${\bar{f}}_{{\text{REL}}_{t+1}}$ is the average relatedness of the target breed with the core set (Wang et al., 2019). It can be seen from this formula that increasing ∆conFGE_*t*_ by minimizing $E\left(f_{{\text{CORE}}_{t+1}}\right)$ results in a decreased relatedness with other breeds and in an increased genetic diversity within the breed. If many breeds are included in the core set, then most of them have a small contribution to the core set. For these breeds, an increase of ∆conFGE_*t*_ results primarily from a reduced average relatedness of the target breed with the core set. In contrast, for breeds with large contributions to the core set, genetic gain in ∆conFGE_*t*_ primarily results from an increased genetic diversity of the breed.

## Handling Introgression

Limited genetic introgression and upgrading with mainstream breeds decrease the level of inbreeding and enable the introduction of advantageous alleles from other populations into the target breed. It can therefore be advisable to introduce a certain amount of foreign genetic material into the population to reduce inbreeding depression, to introduce unique advantageous alleles from other breeds, to increase genetic diversity, and to improve selection response.

Many local breeds, however, were upgraded with mainstream breeds for several decades. As the selection intensity in the local breeds was lower than the selection intensity in the mainstream breeds, the haplotypes from mainstream breeds that entered the local breed decades ago are now economically inferior to the current gene pool of the mainstream breeds. Consequently, upgrading led to sub-optimal performance compared to rotational crossbreeding systems in which the crossbred animals have the same amount of genetic material from mainstream breeds. This is reinforced by the fact that upgrading causes genetic material from these breeds to be eventually present in both parents of each individual, which causes a smaller heterosis effect than a rotation crossbreeding system. Long-term upgrading does not only lead to sub-optimal performance but also leads eventually to the genetic extinction of the local breed because the native genetic background becomes gradually replaced with haplotypes from mainstream breeds. As local breeds were adapted to the environments in which they evolved, the adaptive diversity of the species declines when they lose their native genetic background. An important breeding goal for a breed with historic introgression is therefore to recover the native genetic background or to avoid further introgression.

Whereas some breeding programs aim to recover the native genetic background of a breed, others aim to increase the amount of genetic material from other breeds to get rid of inbreeding depression. In both cases, the target of the breeding program is to reach an optimal value for the native contribution of the breed, which is the proportion of the gene pool that originates from native ancestors. As the native alleles are typically less diverse than the total gene pool of the breed, recovering the native genetic background of a breed increases the level of inbreeding. It is therefore necessary to control the kinship at native haplotype segments to ensure that enough genetic diversity remains in the population after the native genetic background has been recovered. The target parameters for handling introgression are therefore the native contributions and native kinships.

### Native Alleles

A marker allele is native if it belongs to a haplotype segment that originates from a native ancestor, whereby an ancestor is considered native if he was an individual from the target breed and lived before a reference time *t*_0_. The reference time can be chosen in the midpoint between breed formation and the beginning of relevant introgression. We denote the set of native markers from a haplotype **h** as $N_{h}$

**Estimates:** Several methods exist for identifying the native parts of a haplotype. The appropriate method depends on whether gene flow between the target breed and other breeds was unidirectional or bidirectional.

If the direction of gene flow was unidirectional, then the approach of Wang et al. (2017b) can be used, which relies on the segment-based notion of IBD. It requires from each relevant breed *b* a haplotype sample ${ℋ}_{b}$. The relevant breeds are the target breed and the breeds that might have been used for upgrading. As the lengths of IBD segments are used to determine their age, a minimum segment length *m*_0_ needs to be defined. It is chosen such that haplotype segments that entered the population before time *t*_0_ have now an average length smaller than *m*_0_, so $m_{0}=\frac{100}{2g_{0}}\;cM$, where $g_{0}=\frac{t-t_{0}}{L}$ is the number of generations that passed since time *t*_0_ (Browning, 2008). A haplotype **h** from the target breed is considered native at position *m* if the proportion of haplotypes with which **h** is IBD at position *m* is smaller than ϵ in all other breeds. This proportion equals

$${\hat{f}}_{m}(h,\;b)=\bar{f_{\text{SEG}}}(\{h\},\;{ℋ}_{b};\{m\}),$$

so $m\in N_{h}$ if ${\hat{f}}_{m}(h,b)\leq \epsilon$ for all other breeds *b*. The threshold value *є* needs to be specified and may be chosen as ϵ = 0.01.

An alternative approach considers haplotype **h** at position *m* to be native for breed *b*’ if ${\hat{f}}_{m}(h,\;b^{\prime })\geq {\hat{f}}_{m}(h,\;b)$ for all other breeds *b*. This approach, which generalizes Method 2 of Bolormaa et al. (2011), can be used in the case of bidirectional gene flow. It leads, however, in some cases to wrong assignments. In particular, if breed *b*ʹ has been upgraded for a long time with individuals from a specific subpopulation of another breed, then the haplotypes from that subpopulation may eventually be more frequent in breed *b*ʹ than in the other breed, in which case the haplotypes would erroneously be identified as native for breed *b*ʹ.

If no genotype data from other breeds are available, then a haplotype **h** could be considered native at position *m* if, for a window around marker *m*, the vector **h**_*m*_ with gene contents deviates little from the vector with the average gene contents or if the vector **h**_*m*_ can be assigned to the largest cluster. Although this approach has the advantage that no data from other breeds are required, it could lead to wrong results if the amount of introgression in the population is large or if the breeds that were used for upgrading are highly related with the target breed.

Various alternative methods for the identification of native haplotype segments have been developed in addition to the methods described above. Some were applied to identify ancestral introgression in humans, for example, by Racimo et al. (2015). Most of these methods, however, require genome sequences of unadmixed individuals, which are not yet available for most breeds with historic introgression. In the future, they could be obtained from skeletons of animals that lived in the 19th century or earlier.

### Native Kinship

The native kinship between two individuals *i* and *j* is their kinship at native alleles. In mathematical terms,

$$f_{\text{IBD|N}}(i,j)=P(X_{i}\overset{\text{IBD}}{=}Y_{j}|X_{i},\;Y_{j}\in A_{\text{N}})$$

is the conditional probability that two alleles *X*_*i*_,*Y*_*j*_, randomly chosen from individuals *i* and *j* from the same locus are IBD given that they are native. Here, $A_{N}$ is the set of alleles from native founders, and $X_{i}\in A_{N}$ means that allele *X*_*i*_ is IBD with an allele of a native founder. As the native kinship is defined as a conditional probability, it can be expressed as a ratio of two probabilities:

$$f_{\text{IBD|N}}(i,j)=\frac{f_{\text{IBD\&N}}(i,j)}{f_{\text{N}}(i,j)},$$

where *f*_IBD&N_(*i*, *j*) is the probability that alleles *X*_*i*_ and *Y*_*j*_ are both native and IBD, and *f*_N_(*i*, *j*) is the probability that alleles *X*_*i*_ and *Y*_*j*_ are both native. We denote with **f**_IBD&N_ and **f**_N_ the matrices that contain the respective probabilities for all individuals from the current population.

Genetic recovery programs need to keep the average native kinship ${\bar{f}}_{{\text{IBD|N}}_{t}}$ of the population low or, equivalently, the native genetic diversity ${\text{natDiv}}_{t}=1-{\bar{f}}_{{\text{IBD|N}}_{t}}$ high. The expected genetic gain for the native genetic diversity between time *t* and *t* + 1

$$\Delta {\text{natDiv}}_{t}={\bar{f}}_{{\text{IBD|N}}_{t}}-E\left({\bar{f}}_{{\text{IBD|N}}_{t+1}}\right)$$

is negative for most populations because rare alleles get lost due to random genetic drift, but it can be positive for breeding programs that aim to reverse the historical genetic drift. The mean native kinship of the population at time *t* is

$${\bar{f}}_{{\text{IBD|N}}_{t}}=\frac{v_{t}^{T}f_{\text{IBD\&N}}v_{t}}{v_{t}^{T}f_{\text{N}}v_{t}},$$

whereas the expected native kinship of the population at time *t* + 1 is

$$E\left({\bar{f}}_{\text{IBD|}N_{t+1}}\right)=\frac{{\left(r_{0}c+v_{t+1}\right)}^{T}f_{\text{IBD\&N}}(r_{0}c+v_{t+1})+l_{\text{IBD\&N}}(c)}{{\left(r_{0}c+v_{t+1}\right)}^{T}f_{\text{N}}(r_{0}c+v_{t+1})+l_{\text{N}}(c)}.$$

The linear correction terms *l*_IBD&N_(**c**) and *l*_N_(**c**), which are defined in the **Supplementary Material**, account for random genetic drift and for potential sources of bias that can arise when the formulas are used for estimation purposes.

**Estimates:** An approach to estimate the native genetic diversity from pedigrees was proposed by Wellman et al. (2012), who showed for populations with discrete generations that an unbiased estimate of *E* (natDiv_*t*__+ 1_) is obtained when the required matrices **f**_N_ and **f**_IBD&N_ are estimated from pedigree data as

$$\begin{array}{c} {\hat{f}}_{\text{N}}=\frac{1}{2}\left(NC_{t}1^{\text{T}}+1\;NC_{t}^{\text{T}}-11^{\text{T}}+f^{FM}\right),\\ {\hat{f}}_{\text{IBD\&N}}={\hat{f}}_{\text{N}}+f^{M}-f^{FM}, \end{array}$$

where vector **NC**_*t*_ contains the native contributions of the individuals, and matrices **f**^*M*^ and **f**^*FM*^ with

$$\begin{array}{c} f^{M}(i,j)=P(X_{i},\;Y_{j}\notin A_{N}\text{ or }X_{i}\overset{\text{IBD}}{=}Y_{j})\\ f^{FM}(i,j)=P(X_{i},\;Y_{j}\notin A_{N}\text{ or }X_{i},\;Y_{j}\in A_{N}) \end{array}$$

are computed as shown by Wellman et al. (2012). A proof and an algorithm for computing these probabilities can also be found in the **Supplementary Material**. Inserting ${\hat{f}}_{\text{N}}(i,j)$ and ${\hat{f}}_{\text{IBD\&N}}(i,j)$ into Equation 7 provides the pedigree-based native kinship *f*_PED|N_(*i*, *j*) between individuals *i* and *j*.

More accurate estimates can be obtained from marker data. An estimate that relies on the segment-based notion of IBD was proposed by Wang et al. (2017a), in which case the estimates for *f*_N_(*i*, *j*) and *f*_IBD&N_(*i*, *j*) are obtained as follows. The proportion of genome part *M* for which haplotypes **h**_1_ and **h**_2_ are both native is

$${\hat{f}}_{\text{N}}(h_{1},\;h_{2};M)=\frac{\sum_{m\in M\cap N_{h_{1}}\cap N_{h_{2}}}L_{m}}{\sum_{m\in M}\;L_{m}},$$

and the proportion of *M* for which both haplotypes are native and IBD is

$${\hat{f}}_{\text{IBD\&N}}(h_{1},\;h_{2};M)=\frac{\sum_{m\in M\cap \text{IBD}(h_{1},h_{2})\cap N_{h_{1}}\cap N_{h_{2}}}L_{m}}{\sum_{m\in M}L_{m}}$$

The required estimates for individuals *i* and *j*

$${\hat{f}}_{\text{N}}(i,j)={\bar{\hat{f}}}_{\text{N}}(H_{i},\;H_{j};ℳ)$$

$${\hat{f}}_{\text{IBD\&N}}(i,j)=\bar{{\hat{f}}_{\text{IBD\&N}}}(H_{i},\;H_{j};ℳ)$$

are obtained as the average, taken over all pairs of haplotypes, whereby the haplotypes are chosen from the respective individuals. Inserting ${\hat{f}}_{\text{N}}(i,j)$ and ${\hat{f}}_{\text{IBD\&N}}(i,j)$ into Equation 7 provides the segment-based native kinship *f*_SEG|N_(*i*, *j*) between individuals *i* and *j*.

**Discussion:** Although the primary objective of a genetic recovery program is to recover the native genetic background, the removal of foreign haplotype segments from the population may reduce the genetic diversity of the breed. It is important to ensure that enough genetic diversity remains in the breed after the foreign genetic material has been removed to avoid inbreeding depression and to enable future selection response. The mean native kinship tends to increase faster than the conventional kinship because only the purest animals would be used for breeding, which are likely to carry similar native alleles (Wang et al., 2017a). As the kinship and the native kinship become equal after the introgressed genetic material has been removed, and the native kinship increases faster in the first generations of selection, it is important to constrain the rate of increase of the native kinship, whereas constraining the conventional kinship is not required. Hence, the breeding program needs to ensure that the rate of increase of ${\bar{f}}_{\text{IBD}|{\text{N}}_{t}}$ is in accordance with the desired effective size *N*_*e*_. To obtain an effective size of at least *N*_*e*_ for the native alleles, the average native kinship of the population at time *t* + 1 needs to satisfy

(8) $$E\left({\bar{f}}_{{\text{IBD|N}}_{t+1}}\right)\leq 1-\left(1-{\bar{f}}_{{\text{IBD|N}}_{t^{\prime }}}\right){\left(1-\frac{1}{2N_{e}}\right)}^{\frac{t-t^{\prime }+1}{L}}.$$

### Native Contributions

The native contribution of individual *i* is the proportion of its genome, which is native. It equals the probability

$$\eta (i)=P(X_{i}\overset{\text{IBD}}{\in }A_{N})$$

that an allele *X*_*i*_, randomly chosen from the individual is IBD with an allele of a native ancestor. The expected increase of the native contribution between times *t* and *t* + 1 equals

$$\Delta \eta_{t}=E({\bar{\eta }}_{t+1})-{\bar{\eta }}_{t}$$

where ${\bar{\eta }}_{t}$ is the average native contribution in the population at time *t*. The desired value may be positive or negative depending on whether the native genetic background should be recovered, or the inbreeding level should be lowered by new introgression. The values can be calculated as

$${\bar{\eta }}_{t}=v_{t}^{T}\eta_{t}$$

and

$$E({\bar{\eta }}_{t+1})={\left(r_{0}c+v_{t+1}\right)}^{\text{T}}\eta_{t}$$

where ***η***_*t*_ is the vector with native contributions of all individuals.

**Estimates:** The native contribution of an individual can easily be estimated from pedigree data. The pedigree-based native contribution of individual *i*

$$\eta_{\text{PED}}(i)=\sum_{j\in {ℱ}_{N}}\hat{gc}(i,j)$$

is the sum of the genetic contributions individual *i* has from native founders, where *F*_*N*_ is the set of native founders. Thereby, the genetic contribution *gc(i,j)* individual *i* has from ancestor *j* is the proportion of the genome of individual *i* that is contributed by ancestor *j* (James and McBride, 1958). Genetic contributions can be estimated from pedigree data as follows. The contribution individual *i* has from itself is *$\hat{gc}(i,\;i)=1$*, and the contribution individual *i* has from ancestor *j* is

$$\hat{gc}(i,j)=\frac{1}{2}\left(\hat{gc}(s_{i},j)+\hat{gc}(d_{i},j)\right).$$

To be conservative, individuals with unknown pedigrees that were born after reference time are often not considered to be native. More precise estimates that account for Mendelian sampling can be obtained from marker data. As the native proportion of haplotype **h** at genome part *M* is

$$\eta_{\text{SEG}}(h;M)=\frac{\sum_{m\in M\cap N_{h}}\;L_{m}}{\sum_{m\in M}\;L_{m}},$$

the native proportion of haplotype set *ℋ* at genome part *M* is

$$\bar{\eta_{\text{SEG}}}(ℋ;M)=\frac{1}{\left|ℋ\right|}\sum_{h\in ℋ}\eta_{\text{SEG}}(h;M)$$

The segment-based native contribution of individual *i* is therefore

$$\eta_{\text{SEG}}(i)=\bar{\eta_{\text{SEG}}}(H_{i};ℳ).$$

For recovering the native genetic background of a breed, native contributions estimated from pedigrees can only be used for a limited time-span. Thereafter, they must be replaced by marker-based estimates. The reason is that the pedigree-based estimates are the expectations of the true native contributions. However, the true native contributions deviate from their expectations because alleles are transmitted at random from parents to offspring. When pedigree-based estimates are used, then the native contribution cannot be increased beyond the maximum value that is present in the population. Moreover, the pedigree-based estimate cannot be considered a quantitative trait because it has no Mendelian sampling variance. Consequently, recovering the native genetic background requires marker-based estimates of the native contribution.

While recovering the native genetic background of a breed requires marker-based estimates, pedigree-based estimates may be sufficient for breeding programs that aim to reduce inbreeding depression by introgression.

The segment-based estimate of the native contribution can be considered a quantitative trait for which a breeding value can be estimated (Amador et al., 2014). This breeding value for the native contribution can be included as an additional trait in the total merit index.

## General Discussion

The key genetic parameters that have been reviewed in the paper are of different importance for different types of breeding programs. Three main types of breeding programs have been identified in the introduction. The first one aims to maximize the genetic gain of populations that exhibit a sufficient genetic diversity, the second one aims to reduce inbreeding depression in populations that have undergone serious genetic bottlenecks, and the third type aims to increase the value of an endangered breed for conservation. In any case, conflicting breeding objectives need to be balanced. One method to balance conflicting objectives in a breeding program is optimum contribution selection (OCS; Meuwissen, 1997; Grundy et al., 2000; Woolliams et al., 2015), which was originally developed to maximize genetic gain and to restrict the increase of the mean kinship. Advanced OCS methods have been developed, which are implemented in R package optiSel and are able to optimize breeding program with more complex goals (Wellmann, 2019). They enable to compute the optimum number of offspring of each selection candidate such that the population mean of the most important genetic parameter at a future time is optimized, whereas the others are constrained. In the following, the relevance of the genetic parameters and their inclusion in OCS are discussed separately for each type of breeding program.

### Population Management With Focus on Genetic Gain

For genetically diverse breeds that do not rely on funds from conservation programs, the main objective of the breeding program is to keep the breed competitive by maximizing genetic gain for total merit. The common approach is classical OCS proposed by Meuwissen (1997) and Meuwissen and Sonesson (1998), which maximizes the genetic gain of the population until time *t* + 1 and restricts the rate of increase of the mean kinship in accordance with the desired effective population size (Caballero and Toro, 2000). The optimum frequencies of use of breeding animals depend therefore on their breeding values and on the increase in the mean kinship they are causing. Although classical OCS has been shown to be superior in the long term to alternative existing approaches such as truncation selection, it is not the optimum method for long-term population management. The reason is that classical OCS maximizes the expected genetic gain of the population until the next evaluation time *t* + 1, which is not optimal because some rewards for choosing a particular mate come delayed.

There are different types of delayed rewards. This paragraph discusses possibilities to account for delayed rewards in the OCS framework. First, selection candidates that cause high Mendelian sampling variances have more likely some top-ranking offspring, which qualifies these offspring for broad use as elite sires or dams, so their use increases the mean breeding value in the generation after next. Accounting for Mendelian sampling variances has therefore a delayed effect on the mean breeding value of the population. Classical OCS, which maximizes the mean breeding value at time *t* + 1 cannot account for this. Although accounting for Mendelian sampling variances has a delayed effect on the population mean, it has an immediate effect on the probability to breed top-ranking animals. A straightforward approach is therefore to maximize not the mean breeding value but the probability to breed top-ranking individuals. This is roughly achieved when the computation of optimum contributions is based on the index from Equation 2. This improved approach favors individuals for breeding, which have simultaneously a high total merit index and a high Mendelian sampling variance. A further delayed reward may occur if a breeder does not use one of the first offspring of an elite animal but waits for his best offspring. Using one of the first offspring may be an inferior breeding strategy because this approach would accumulate his genes in the population. As a consequence, close relatives with even higher breeding values cannot be intensely used for breeding in the future because this would substantially increase the mean kinship of the population. If the probability is high that superior half-sibs become available in the future, it may be advisable to penalize the use of an early offspring of an elite animal by adding a penalty term to his breeding value. This could be done until a substantial proportion of his half-sibs is available. In summary, the recommended strategy for OCS is not to maximize total merit but to maximize the index from Equation 2 with an additional temporary penalty term for early offspring from elite animals.

To avoid problems with inbreeding depression and to ensure long-term response to selection, the rate of increase of the mean kinship needs to be restricted in accordance with the desired effective population size. Equation 5 is used to compute the maximum permissible kinship of the population at the next evaluation time *t* + 1, whereas the expected value at time *t* + 1 is computed with Equation 4. This equation contains a correction term that accounts for some potential sources of bias. Omitting the correction term would unduly penalize the use of selection candidates from age classes with small sample size because their genes would appear to be already over-represented in the population. The required kinships are traditionally estimated from pedigrees. This has not only the disadvantage that the estimates are less accurate than segment-based estimates but also that the optimum contributions can be strongly skewed by individuals with short pedigrees. Individuals with short pedigrees are favored for breeding because they appear to be less related with the population (Mucha and Windig, 2009). Different approaches exist to overcome this problem. One possibility is to truncate all pedigrees such that they refer to a younger base population and to exclude individuals with an insufficient number of equivalent complete generations in the pedigree from breeding. Alternatively, a constraint can be applied which poses a lower bound for the average number of equivalent complete generations in the pedigrees of the offspring. Third, the kinship constraint could be replaced by a constraint for the pedigree-based native kinship, whereby founders born after some reference date *t*_0_ are considered to be non-native. In this case, the average kinship at alleles originating from founders born before *t*_0_ is restricted, whereas alleles originating from founders born thereafter are ignored. The recommended approach, however, is not to use pedigree-based estimates, but segment-based estimates that are obtained from a panel with at least 50K markers.

An optimal strategy for population management does not only determine the optimum number of offspring of each selection candidate but also optimizes the mate allocation. To maximize the probability of breeding top-ranking individuals, mates could be allocated such that the index from Equation 1 is maximized. This practice, however, seems to have little effect on long-term genetic gain, so alternative strategies for mate allocation could be superior. An alternative strategy is to breed for high Mendelian sampling variances. Individuals have high Mendelian sampling variances if their parents are unrelated. A promising strategy is therefore to mate unrelated individuals, which is achieved when the objective of mate allocation is to minimize the average inbreeding coefficient of the offspring.

### Population Management With Focus on Inbreeding Depression

Several domestic breeds have experienced serious genetic bottlenecks, which decreased their fitness and fertility. Reasons for these bottlenecks were the founder event (Wellmann and Pfeiffer, 2009), the overuse of sires from a small number of popular breeders (Wellmann and Bennewitz, 2011b), and small historical population sizes (Kettunen et al., 2017). The bottlenecks increased the probability that recessive deleterious alleles are homozygous, which resulted in inbreeding depression and, in particular, in an increased prevalence of genetic disorders. The main objective of breeding programs for these breeds is to reduce inbreeding depression, which can be achieved by minimizing the mean kinship of the population and by the purging of genetic load. Both approaches can be combined into a single breeding strategy.

The purging approach aims to decrease the frequencies of deleterious alleles in the population. As inbreeding is usually due to the deleterious effects of many alleles, all individuals can expected to be carriers of some deleterious alleles. Excluding all carriers from breeding is therefore not an option. One possibility to handle this problem is to estimate for all mutations the probability to be deleterious and the expected effect size. These estimates can be incorporated into a breeding value for genetic load, which becomes part of the total merit index. In addition, the most deleterious mutations could be reversed by genome editing.

The second approach aims to increase the heterozygosity of the population. As all individuals of the population are related with each other, this can only partly be achieved by mating the least related individuals. The main goal of a breeding program for these breeds is to reduce the mean kinship of the population. As pedigree-based kinship estimates are unable to capture Mendelian sampling effects, their use would lead after a few generations to a selection plateau, at which no further reduction of the mean kinship can be achieved. This can be avoided by the use of segment-based kinship estimates. The reduction of the mean kinship that can be achieved with segment-based estimates depends on the average number of pairwise non-IBD founder alleles that are still segregating in the population. If this number is too low, which is, for example, the case for the Kromfohrländer breed and the Lundehund (Wellmann and Pfeiffer, 2009; Kettunen et al., 2017), then the introduction of genetic material from other breeds is inevitable for recovering fitness and fertility. However, genetic introgression with other breeds should be limited to the necessary minimum to preserve genetic uniqueness of the breed. To achieve a strong reduction of the average kinship with a limited amount of introgression, the individuals that enter the population should be genetically diverse and numerous, but each of them should make only a small contribution to the population.

The recommended approach is therefore to minimize the segment-based kinship of the breed with OCS, to restrict the native contribution at its desired value, to incorporate a breeding value for genetic load into the total merit index, and to impose an annually increasing lower bound for the mean total merit of the population.

### Population Management With Focus on Conservation Value

As endangered breeds compete for funds from conservation programs, a primary breeding goal for these breeds is to increase their value for conservation. As outlined in the introduction, this can be achieved by selecting individuals with rare haplotype segments for breeding, which increases the contribution of the breed to the genetic diversity of the species and makes it more dissimilar to other breeds. This can be done either by recovering the native genetic background of a breed with historic introgression or by accumulating rare haplotype segments in the breed regardless of their origin.

If the goal of the breeding program is to accumulate rare haplotype segments regardless of their origin, then the FGE can be maximized that is contributed by the breed to the gene pool of the species. As described above, this can be achieved with an objective function that simultaneously aims to increase the genetic diversity of the breed and at reducing the average relatedness of the breed with other breeds (Equation 6). The weight *b*_*k*_ given to the within breed genetic diversity is high if the gene pool of the breed overlaps little with the gene pool of other breeds. If the genetic overlap is high, i.e. the breed does not currently contribute to the genetic diversity of the species, then the approach is equivalent to minimizing the relatedness of the breed with other breeds. Minimizing the average relatedness with other breeds was already studied by Amador et al. (2013) and requires to restrict the rate of increase of the mean kinship of the breed. The application of this approach to breeds with historic introgression would prioritize the removal of haplotype segments from the local breed that are frequent in mainstream breeds because these segments contribute most to the kinship between breeds. Prioritizing their removal, however, may be undesirable because they carry more likely important QTL.

The alternative approach is to recover the native genetic background of the breed. Different objective functions have been proposed for this purpose. A common approach is to maximize either the native contribution or a total merit index that incorporates the native contribution. Wang et al. (2019) proposed another promising strategy, which is to maximize the native FGE of the breed. The native FGE of the breed is defined as

$${\text{natFGE}}_{t}=\frac{{\bar{\eta }}_{t}}{2{\bar{f}}_{{\text{IBD|N}}_{t}}}$$

This genetic parameter measures the native proportion ${\bar{\eta }}_{t}$ of a founder gene pool whose genetic diversity is equal to the native genetic diversity of the breed. Note that this parameter differs from the parameter NGE defined by Wellmann et al. (2012).

The native FGE has several nice properties. The native FGE of a breed without introgression equals the FGE of the breed, and the native FGE converges to 0, when all native alleles become replaced with alleles from other breeds. This parameter combines the native contribution of the breed and its native kinship in a meaningful way such that a breeding program that increases the native FGE tends to increase both the native contribution and the native diversity of the population. Maximizing the native FGE of a breed in a genetic recovery program is thus an interesting alternative to maximizing the native contribution of the breed.

As the conventional kinship of the population equals the native kinship after the native genetic background has been recovered, the native kinship determines the genetic diversity that can be preserved in the population. The breeding program needs therefore to ensure that the native kinship does not increase faster than required for the maintenance of the desired effective size. In a population with a small effective size, the positive effect of making the breed genetically more dissimilar to other breeds could become overcompensated by the negative effect resulting from lost genetic diversity (see Equation 6). An effective population size of more than 100 may be needed to balance both effects, which ensures that the contribution of the breed to the genetic diversity of the species does not decline in the course of the recovery program. For very small local breeds consisting of about 400 individuals (200 male and 200 female selection candidates), the recovery of the native genetic background requires many generations if an effective size of 100 should be maintained. The recovery proceeds much faster in populations consisting of 1000 individuals because a higher selection intensity can be achieved (Wang et al., 2019). The complete removal of foreign genetic material, however, can only be recommended if the native alleles have a sufficiently high genetic diversity in the current population because the breeding program would otherwise result in high inbreeding coefficients and inbreeding depression. If simple policies for genetic recovery are used, then, as demonstrated by the Abondance breed, even a small recovery can lead to relevant inbreeding (Danchin-Burge et al., 2012). In addition, a genetic recovery program can only be recommended if the funds from conservation programs compensate for the reduced genetic gain in total merit.

## Author Contributions

RW did the mathematical part and wrote the manuscript. JB contributed to the literature survey and the writing of the manuscript.

## Funding

Thestudy was supported by the German Research Foundation (Deutsche Forschungsgemeinschaft, DFG).

## Conflict of Interest Statement

: The authors declare that the research was conducted in the absence of any commercial or financial relationships that could be construed as a potential conflict of interest.
