# Supplementary material for: Key Genetic Parameters for Population Management
Source: Front Genet. 2019 Aug 16;10:667. doi: 10.3389/fgene.2019.00667 (PMC6707806; doi:10.3389/fgene.2019.00667)
Supplement: Supplementary file 1 [file DataSheet_1.pdf]

Electronic Appendix

Robin Wellmann

May 16, 2019

Contents

1 Notations **2**

2 A linear index that accounts for Mendelian sampling variance **3**

3 Mean kinship in the population **6**

3.1 Expected value at time t 6

3.2 Expected value at time $t+1$ 9

3.3 Estimate for time $t+1$ 16

4 Mean native kinship in the population **22**

4.1 Expected value at time t 22

4.2 Estimate for time $t+1$ 24

4.3 Pedigree-based estimate of native kinship 27

5 Number of parent-offspring pairs in the population at time t **29**

This appendix of the paper "Tools for Optimal Population Management" includes the proofs of the formulas.

1 Notations

The population at time t is denoted as $\mathcal{P}_t$. It is divided into q different classes

$$\mathcal{P}_t = \cup_{s=1}^{q} \mathcal{P}_s^t.$$

These classes can be sex classes (in which case $q = 2$), age classes, or age×sex classes. Class s has contribution r_s^t to the population, so

$$\sum_{s=1}^{q} r_s^t = 1.$$

The probability that an individual randomly chosen from the population belongs to class s is $P(i \in \mathcal{P}_s^t) = r_s^t$. The number of individuals belonging to this class is $N_s^t = r_s^t N$, where N is the population size.

The population size N is assumed to be constant. The contribution of class $s = 1, ..., q$ to the population at time $t+1$ is r_s^{t+1}, so the number of individuals in this class at time $t+1$ is $N_s^{t+1} = r_s^{t+1} N$.

The set of individuals from class s that are still in the population at time $t+1$ is $\mathcal{P}_s^{t+1}$. Some individuals that were alive at time t no longer contribute to the population at time $t+1$, e.g. because they are no longer fertile. Hence,

$$\mathcal{P}_s^{t+1} \subseteq \mathcal{P}_s^t \text{ and } r_s^{t+1} \leq r_s^t.$$

The offspring $\mathcal{O}_{t+1}$ consisting of the individuals to be born at time $t+1$ have contribution

$$r_0 = 1 - \sum_{s=1}^{q} r_s^{t+1}$$

to the population at time $t+1$. The number of individuals in this class is $N_0 = r_0 N$. The individuals in the population at time $t+1$ that were already born at time t are denoted as

$$\mathcal{I}_{t+1} = \cup_{s=1}^{q} \mathcal{P}_s^{t+1},$$

so the population at time $t+1$ is

$$\mathcal{P}_{t+1} = \mathcal{O}_{t+1} \cup \mathcal{I}_{t+1}.$$

The vector containing the contributions of all classes to the population at time t is

$$\mathbf{r}_t = (r_1^t, ..., r_q^t)^T,$$

whereas the vector containing the contributions these classes have to the population at time $t+1$ is

$$\mathbf{r}_{t+1} = (r_1^{t+1}, ..., r_q^{t+1})^T.$$

2 A linear index that accounts for Mendelian sampling variance

Take $\overline{BV}_t^s$ to be an estimate for the average breeding value for total merit for breeding animals of sex s at time t.

Let BV_i be the breeding value of selection candidate i and MV_i its Mendelian sampling variance. Let $s \in \{f, m\}$ be the sex of individual i, $\overline{s}$ the opposite sex, and s' the desired sex of the offspring. Let $\kappa = 1$ if the planned mating should produce a female (i.e. $s' = f$) and $\kappa = 0$ otherwise. Let $g_{s'} > 0$ be the time that passes until the offspring of the mating could be used for breeding.

In order to construct an index that accounts for Mendelian sampling and is linear in $\mu_{i\circ}$ and $\sigma_{i\circ}$, a linear approximation of the function

$$f_{s'}(\mu_{i\circ}, \sigma_{i\circ}) = \frac{\mu_{i\circ} - \overline{BV}_{t+g_{s'}}^{s'}}{\sigma_{i\circ}}$$

at $\mu_{\circ\circ} = \frac{\overline{BV}_t^m + \overline{BV}_t^f}{2}$ and $\sigma_{\circ\circ} = \sqrt{\frac{\sigma_{At}^2}{2}}$ is needed, where σ_{At}^2 is the additive variance for total merit in the population at time t. The linear approximation is

$$\hat{f}_{s'}(\mu_{i\circ}, \sigma_{i\circ}) = \frac{1}{\sigma_{\circ\circ}} \left(\mu_{\circ\circ} - 2\overline{BV}_{t+g_{s'}}^{s'} + \mu_{i\circ} + \sigma_{i\circ} \frac{\lambda_{s'}}{\sqrt{2}} \right),$$

where $\lambda_{s'} = \sqrt{2} \frac{\overline{BV}_{t+g_{s'}}^{s'} - \mu_{\circ\circ}}{\sigma_{\circ\circ}}$. This function, evaluated at $\mu_{i\circ} = \frac{\overline{BV}_{\overline{s}t} + BV_i}{2}$ and $\sigma_{i\circ} = \sqrt{\mathrm{MV}_i + \frac{\sigma_{At}^2}{4}}$, is a linear transformation of

$$I_i = BV_i + \lambda_{s'}\sqrt{2\mathrm{MV}_i + \frac{\sigma^2_{At}}{2}}.$$

The weight $\lambda_{s'}$ can be expressed as

$$\lambda_{s'} = \frac{2\Delta G_{s'} + (-1)^{\kappa}\Delta S_{s'}}{\sigma_{At}}.$$

It depends on the desired sex s' of the offspring. In this formula, $\Delta G_{s'} = \overline{BV}^{s'}_{t+g_{s'}} - \overline{BV}^{s'}_{t}$ is the genetic gain for total merit per generation (i.e. within $g_{s'}$ years), and $\Delta S_{s'} = \overline{BV}^{m}_{t} - \overline{BV}^{f}_{t}$ is the average difference in the breeding value for total merit between the breeding males and breeding females that are potential parents for offspring of sex s'.

Proof:

We have

$$\begin{aligned}
\hat{f}_{s'}(\mu_{i\circ}, \sigma_{i\circ}) &= f_{s'}(\mu_{\circ\circ}, \sigma_{\circ\circ}) + (\mu_{i\circ} - \mu_{\circ\circ})\frac{d}{d\mu_{i\circ}} f_{s'}(\mu_{\circ\circ}, \sigma_{\circ\circ}) + (\sigma_{i\circ} - \sigma_{\circ\circ})\frac{d}{d\sigma_{i\circ}} f_{s'}(\mu_{\circ\circ}, \sigma_{\circ\circ}) \\
&= \frac{\mu_{\circ\circ} - \overline{BV}^{s'}_{t+g_{s'}}}{\sigma_{\circ\circ}} + (\mu_{i\circ} - \mu_{\circ\circ})\frac{1}{\sigma_{\circ\circ}} - (\sigma_{i\circ} - \sigma_{\circ\circ})\frac{\mu_{\circ\circ} - \overline{BV}^{s'}_{t+g_{s'}}}{\sigma^2_{\circ\circ}} \\
&= \frac{1}{\sigma_{\circ\circ}}\left(\mu_{\circ\circ} - \overline{BV}^{s'}_{t+g_{s'}} + \mu_{i\circ} - \mu_{\circ\circ} - (\sigma_{i\circ} - \sigma_{\circ\circ})\frac{\mu_{\circ\circ} - \overline{BV}^{s'}_{t+g_{s'}}}{\sigma_{\circ\circ}}\right) \\
&= \frac{1}{\sigma_{\circ\circ}}\left(\mu_{\circ\circ} - \overline{BV}^{s'}_{t+g_{s'}} + \mu_{i\circ} - \mu_{\circ\circ} + \mu_{\circ\circ} - \overline{BV}^{s'}_{t+g_{s'}} - \sigma_{i\circ}\frac{\mu_{\circ\circ} - \overline{BV}^{s'}_{t+g_{s'}}}{\sigma_{\circ\circ}}\right) \\
&= \frac{1}{\sigma_{\circ\circ}}\left(\mu_{\circ\circ} - 2\overline{BV}^{s'}_{t+g_{s'}} + \mu_{i\circ} + \sigma_{i\circ}\frac{\overline{BV}^{s'}_{t+g_{s'}} - \mu_{\circ\circ}}{\sigma_{\circ\circ}}\right) \\
&= \frac{1}{\sigma_{\circ\circ}}\left(\mu_{\circ\circ} - 2\overline{BV}^{s'}_{t+g_{s'}} + \mu_{i\circ} + \sigma_{i\circ}\frac{\lambda_{s'}}{\sqrt{2}}\right)
\end{aligned}$$

Thus, for $\mu_{i\circ} = \frac{\overline{BV}_{\bar{s}t} + BV_i}{2}$ and $\sigma_{i\circ} = \sqrt{\text{MV}_i + \frac{\sigma_{At}^2}{4}}$ we have

$$\begin{aligned}
\hat{f}_{s'}(\mu_{i\circ}, \sigma_{i\circ}) &= \frac{1}{2\sigma_{\circ\circ}} \left(2\mu_{\circ\circ} - 4\overline{BV}_{t+g_{s'}}^{s'} + 2\mu_{i\circ} + \sigma_{i\circ}\sqrt{2}\lambda_{s'} \right) \\
&= \frac{1}{2\sigma_{\circ\circ}} \left(2\mu_{\circ\circ} - 4\overline{BV}_{t+g_{s'}}^{s'} + \overline{BV}_t^{\bar{s}} + BV_i + \sigma_{i\circ}\sqrt{2}\lambda_{s'} \right) \\
&= \frac{1}{2\sigma_{\circ\circ}} \left(2\mu_{\circ\circ} - 4\overline{BV}_{t+g_{s'}}^{s'} + \overline{BV}_t^{\bar{s}} + BV_i + \lambda_{s'}\sqrt{2}\sqrt{\text{MV}_i + \frac{\sigma_{At}^2}{4}} \right) \\
&= \frac{1}{2\sigma_{\circ\circ}} \left(2\mu_{\circ\circ} - 4\overline{BV}_{t+g_{s'}}^{s'} + \overline{BV}_t^{\bar{s}} + I_i \right).
\end{aligned}$$

Furthermore, since

$$\begin{aligned}
\mu_{\circ\circ} &= \frac{\overline{BV}_t^{m} + \overline{BV}_t^{f}}{2} \\
&= \frac{2\overline{BV}_t^{m} - \Delta S_{s'}}{2} \\
&= \overline{BV}_t^{m} - \frac{\Delta S_{s'}}{2} \\
&= \overline{BV}_t^{s'} + 1_f(s')\Delta S_{s'} - \frac{\Delta S_{s'}}{2} \\
&= \overline{BV}_t^{s'} - \left(\frac{1}{2} - 1_f(s') \right) \Delta S_{s'} \\
&= \overline{BV}_t^{s'} - \frac{(-1)^{\kappa}}{2} \Delta S_{s'}
\end{aligned}$$

and

$$\Delta G_{s'} = \overline{BV}_{t+g_{s'}}^{s'} - \overline{BV}_t^{s'},$$

we have

$$
\begin{aligned}
\lambda_{s'} &= \sqrt{2}\frac{\overline{BV}^{s'}_{t+g_{s'}} - \mu_{\circ\circ}}{\sigma_{\circ\circ}} \\
&= \frac{2}{\sqrt{2}}\frac{\overline{BV}^{s'}_{t+g_{s'}} - \overline{BV}^{s'}_{t} + \frac{(-1)^\kappa}{2}\Delta S_{s'}}{\sigma_{\circ\circ}} \\
&= \frac{2}{\sqrt{2}}\frac{\Delta G_{s'} + \frac{(-1)^\kappa}{2}\Delta S_{s'}}{\sigma_{\circ\circ}} \\
&= \frac{1}{\sqrt{2}}\frac{2\Delta G_{s'} + (-1)^\kappa\Delta S_{s'}}{\sqrt{\frac{\sigma^2_{At}}{2}}} \\
&= \frac{2\Delta G_{s'} + (-1)^\kappa\Delta S_{s'}}{\sigma_A}.
\end{aligned}
$$

3 Mean kinship in the population

3.1 Expected value at time t

The mean kinship in the population is the probability that two alleles X_i, Y_j chosen from randomly selected individuals i,j are identical (by descent). It is well known that the mean kinship is

$$f_{IBD}(\mathcal{P}_t) = P_t(X_i \overset{IBD}{=} Y_j) \;=\; \mathbf{v}_t^T \mathbf{f}_t \mathbf{v}_t,$$

where $\mathbf{f}_t$ is the matrix with pairwise kinships of the individuals and $v_{ti} = \frac{r_s^t}{N_s^t}$ equals $\frac{1}{N}$. However, using the above formula to estimate the mean kinship from a sample provides a biased estimate because the probability of sampling alleles from the same individual increases as the sample size decreases, and the sample may contain a different proportion of parent-offspring pairs than the population.

An alternative formula for $f_{IBD}(\mathcal{P}_t)$ from which a less biased estimator can be obtained is

$$f_{IBD}(\mathcal{P}_t) \;=\; \mathbf{r}_t^T \left(\tilde{\mathbf{f}}_t + \Delta\tilde{\mathbf{f}}_t\right) \mathbf{r}_t, \qquad (1)$$

where $q \times q$-matrix $\tilde{\mathbf{f}}_t$ contains the average kinships $\tilde{f}_t(s_1, s_2)$ between individuals from classes s_1 and s_2, whereby self-kinships and parent-offspring pairs

are excluded. It is obtained from matrix $\mathbf{f}_t$, containing pairwise kinships of the individuals from population $\mathcal{P}_t$.

The second matrix corrects for the bias that would arise from excluding self-kinships and parent-offspring pairs. Hence, matrix $\Delta\tilde{\mathbf{f}}_t$ is defined as

$$\Delta\tilde{f}_{t,s_1s_2} = \frac{N_{s_1,s_2}}{N_{s_1}N_{s_2}}\left(\tilde{F}_{t,s_1s_2} - \tilde{f}_{t,s_1s_2}\right),$$

where $q \times q$ - matrix $\tilde{\mathbf{F}}_t$ contains in the diagonal the average self-kinships of individuals from each class s, and in the off-diagonals the average kinships of parent-offspring pairs. Moreover, N_{s_1,s_2} for $s_1 \neq s_2$ is the number of parent-offspring pairs in the population for which one individual belongs to $\mathcal{P}^t_{s_1}$ and the other individual belongs to $\mathcal{P}^t_{s_2}$, and $N_{s,s} = N^t_s$ is the number of individuals in class s.

The above formula can be used to estimate the mean kinship from a sample as

$$\hat{P}_t(X_i \overset{IBD}{=} Y_j) = \mathbf{r}_t^T\left(\widehat{\tilde{\mathbf{f}}}_t + \widehat{\Delta\tilde{\mathbf{f}}_t}\right)\mathbf{r}_t, \qquad (2)$$

where $\widehat{\tilde{\mathbf{f}}}_t$ and $\widehat{\Delta\tilde{\mathbf{f}}_t}$ are computed from the sample in the same way as $\tilde{\mathbf{f}}_t$ and $\Delta\tilde{\mathbf{f}}_t$ were computed from the population. A formula for estimating the number N_{s_1,s_2} of parent-offspring pairs in the populatiuon is derived in the last section.

Proof:

For brevity we write $\mathcal{P}_s = \mathcal{P}^t_s$ in this proof. Let $\mathcal{R}$ be a set consisting of all parent-offspring pairs and all pairs (i,i) from the population. That is,

$$(i,j) \in \mathcal{R} \;\Leftrightarrow\; i = j, \text{ or } j = sire_i, \text{ or } i = sire_j, \text{ or } j = dam_i, \text{ or } i = dam_j.$$

where $sire_i$ is the sire of i and dam_i is the dam of i. We have

$$
\begin{aligned}
& P_t(X_i \overset{IBD}{=} Y_j) \\
= & \sum_{s_1,s_2} P(i \in \mathcal{P}_{s_1}, j \in \mathcal{P}_{s_2}, (i,j) \notin \mathcal{R}) P(X_i = Y_j | i \in \mathcal{P}_{s_1}, j \in \mathcal{P}_{s_2}, (i,j) \notin \mathcal{R}) \\
+ & \sum_{s_1,s_2} P(i \in \mathcal{P}_{s_1}, j \in \mathcal{P}_{s_2}, (i,j) \in \mathcal{R}) P(X_i = Y_j | i \in \mathcal{P}_{s_1}, j \in \mathcal{P}_{s_2}, (i,j) \in \mathcal{R}) \\
= & \sum_{s_1,s_2} P((i,j) \notin \mathcal{R} | i \in \mathcal{P}_{s_1}, j \in \mathcal{P}_{s_2}) P(i \in \mathcal{P}_{s_1}, j \in \mathcal{P}_{s_2}) P(X_i = Y_j | i \in \mathcal{P}_{s_1}, j \in \mathcal{P}_{s_2}, (i,j) \notin \mathcal{R}) \\
+ & \sum_{s_1,s_2} P((i,j) \in \mathcal{R} | i \in \mathcal{P}_{s_1}, j \in \mathcal{P}_{s_2}) P(i \in \mathcal{P}_{s_1}, j \in \mathcal{P}_{s_2}) P(X_i = Y_j | i \in \mathcal{P}_{s_1}, j \in \mathcal{P}_{s_2}, (i,j) \in \mathcal{R}) \\
= & \sum_{s_1,s_2} \left(1 - \frac{N_{s_1,s_2}}{N_{s_1} N_{s_2}}\right) r^t_{s_1} r^t_{s_2} P(X_i = Y_j | i \in \mathcal{P}_{s_1}, j \in \mathcal{P}_{s_2}, (i,j) \notin \mathcal{R}) \\
+ & \sum_{s_1,s_2} \frac{N_{s_1,s_2}}{N_{s_1} N_{s_2}} r^t_{s_1} r^t_{s_2} P(X_i = Y_j | i \in \mathcal{P}_{s_1}, j \in \mathcal{P}_{s_2}, (i,j) \in \mathcal{R})
\end{aligned}
$$

Thus,

$$
\begin{aligned}
P_t(X_i \overset{IBD}{=} Y_j) &= \sum_{s_1,s_2} r^t_{s_1} r^t_{s_2} P(X_i = Y_j | i \in \mathcal{P}_{s_1}, j \in \mathcal{P}_{s_2}, (i,j) \notin \mathcal{R}) \\
&+ \sum_{s_1,s_2} r^t_{s_1} r^t_{s_2} \frac{N_{s_1,s_2}}{N_{s_1} N_{s_2}} P(X_i = Y_j | i \in \mathcal{P}_{s_1}, j \in \mathcal{P}_{s_2}, (i,j) \in \mathcal{R}) \\
&- \sum_{s_1,s_2} r^t_{s_1} r^t_{s_2} \frac{N_{s_1,s_2}}{N_{s_1} N_{s_2}} P(X_i = Y_j | i \in \mathcal{P}_{s_1}, j \in \mathcal{P}_{s_2}, (i,j) \notin \mathcal{R}) \\
&= \sum_{s_1,s_2} r^t_{s_1} r^t_{s_2} \tilde{f}_{t,s_1 s_2} + \sum_{s_1,s_2} r^t_{s_1} r^t_{s_2} \frac{N_{s_1,s_2}}{N_{s_1} N_{s_2}} \left(\tilde{F}_{t,s_1 s_2} - \tilde{f}_{t,s_1 s_2}\right) \\
&= \mathbf{r}_t^T \tilde{\mathbf{f}}_t \mathbf{r}_t + \mathbf{r}_t^T \Delta \tilde{\mathbf{f}}_t \mathbf{r}_t
\end{aligned}
$$

where

$$
\begin{aligned}
\tilde{f}_{t,s_1 s_2} &= P(X_i = Y_j | i \in \mathcal{P}_{s_1}, j \in \mathcal{P}_{s_2}, (i,j) \notin \mathcal{R}), \\
\tilde{F}_{t,s_1 s_2} &= P(X_i = Y_j | i \in \mathcal{P}_{s_1}, j \in \mathcal{P}_{s_2}, (i,j) \in \mathcal{R}).
\end{aligned}
$$

Since no parent-offspring pair can be in the same age class, we have

$$
\begin{aligned}
\tilde{F}_{t,ss} &= P(X_i = Y_j | i \in \mathcal{P}_s, j \in \mathcal{P}_s, i = j) \\
&= P(X_i = Y_i | i \in \mathcal{P}_s) \\
&= \frac{1}{N_s} \sum_{i \in \mathcal{P}_s} P(X_i = Y_i) \\
&= \frac{1}{N_s} \sum_{i \in \mathcal{P}_s} f_{t,ii} \\
&\approx \frac{1}{2}
\end{aligned}
$$

This can be estimated from a sample as the average of the diagonal elements from matrix $\widehat{\mathbf{f}}_t$ corresponding to individuals from class s. For $s_1 \neq s_2$, the value is

$$
\tilde{F}_{t,s_1 s_2} = \frac{1}{N_{s_1 s_2}} \sum_{(i,j) \in \mathcal{R} \cap \mathcal{P}_{s_1} \times \mathcal{P}_{s_2}} f_{t,ij} \approx \frac{1}{4},
$$

where the sum is over all parent-offspring pairs from classes s_1 and s_2.

□

3.2 Expected value at time $t + 1$

a) The average kinship in the population at time $t + 1$ is

$$
f_{IBD}(\mathcal{P}_{t+1}) = r_0^2 f_{IBD}(\mathcal{O}_{t+1}) + 2r_0(1 - r_0) f_{IBD}(\mathcal{I}_{t+1}, \mathcal{O}_{t+1}) + (1 - r_0)^2 f_{IBD}(\mathcal{I}_{t+1}),
$$

where

$$
f_{IBD}(\mathcal{O}_{t+1}) = P(X_i = Y_j | i, j \in \mathcal{O}_{t+1})
$$

is the probability that two alleles randomly chosen from the offspring are IBD,

$$
f_{IBD}(\mathcal{I}_{t+1}, \mathcal{O}_{t+1}) = P(X_i = Y_j | i \in \mathcal{I}_{t+1}, j \in \mathcal{O}_{t+1})
$$

is the probability that two alleles are IBD if one is chosen from the offspring and one is chosen from individuals that were already born at time t, and

$$
f_{IBD}(\mathcal{I}_{t+1}) = P(X_i = Y_j | i, j \in \mathcal{I}_{t+1})
$$

is the probability that two alleles are IBD that are randomly chosen from individuals that were already born at time t.

b) The expected mean kinship in the offspring that will be born at time $t+1$ is

$$f_{IBD}(\mathcal{O}_{t+1}) = \mathbf{c}^T \mathbf{f}_t \mathbf{c} + \frac{1 - \mathbf{c}^T d(\mathbf{f}_t)}{2N_0},$$

where matrix $\mathbf{f}_t$ contains pairwise kinships of individuals from the population at time t, vector $d(\mathbf{f}_t)$ contains the diagonal elements of matrix $\mathbf{f}_t$, and vector $\mathbf{c}$ contains the genetic contributions of all individuals from the population at time t to the offspring that will be born at time $t+1$.

c) The expected mean kinship of the individuals that were already born at time t and are still in the population at time $t+1$ is

$$f_{IBD}(\mathcal{I}_{t+1}) = \frac{\mathbf{r}_{t+1}^T \left(\overline{\mathbf{f}}_t + \Delta_1 \overline{\mathbf{f}}_t\right) \mathbf{r}_{t+1}}{(1-r_0)^2},$$

where $q \times q$-matrix $\overline{\mathbf{f}}_t$ contains the average kinships $\overline{f}_t(s_1, s_2)$ between individuals from classes s_1 and s_2, whereby self-kinships and parent-offspring pairs are included. The $q \times q$ diagonal matrix $\Delta_1 \overline{\mathbf{f}}_t$ has diagonal elements

$$\Delta_1 \overline{f}_{t,ss} = \left(\frac{1}{N_s^{t+1}} - \frac{1}{N_s^t}\right)(\tilde{F}_{t,s} - \tilde{f}_{t\,ss}).$$

It accounts for the fact that some individuals that were in the population at time t are no longer in the population at time $t+1$, so the probability of sampling two alleles from the same individual increases within each class.

d) The mean kinship between the offspring and the individuals that were already born at time t is

$$f_{IBD}(\mathcal{I}_{t+1}, \mathcal{O}_{t+1}) = \frac{\mathbf{c}^T \overline{\mathbf{f}}^t_{N \times q} \mathbf{r}_{t+1}}{1 - r_0},$$

where $\overline{\mathbf{f}}^t_{N \times q}$ is a $N \times q$-matrix containing in row k and column s the average kinship of individual k from the population at time t with the individuals from age class s.

Proofs

Proof of a)

The set of individuals from class s still belonging to the population at time $t+1$ is denoted as $\mathcal{P}_s^{t+1}$.

The probability that an individual randomly chosen from the population at time $t+1$ was not yet born at time t is $P(i \in \mathcal{O}_{t+1}) = r_0$, whereas the probability that it was born at time t or earlier is $P(i \in \mathcal{I}_{t+1}) = 1 - r_0$. Thus, the mean kinship at time $t+1$ is

$$\begin{aligned}
f_{IBD}(\mathcal{P}_{t+1}) &= P_{t+1}(X_i = Y_j) \\
&= P(i,j \in \mathcal{O}_{t+1})P(X_i = Y_j | i,j \in \mathcal{O}_{t+1}) \\
&+ P(i \in \mathcal{O}_{t+1}, j \in \mathcal{I}_{t+1})P(X_i = Y_j | i \in \mathcal{O}_{t+1}, j \in \mathcal{I}_{t+1}) \\
&+ P(i \in \mathcal{I}_{t+1}, j \in \mathcal{O}_{t+1})P(X_i = Y_j | i \in \mathcal{I}_{t+1}, j \in \mathcal{O}_{t+1}) \\
&+ P(i,j \in \mathcal{I}_{t+1})P(X_i = Y_j | i,j \in \mathcal{I}_{t+1}) \\
&= r_0^2 f_{IBD}(\mathcal{O}_{t+1}) + 2r_0(1-r_0) f_{IBD}(\mathcal{I}_{t+1}, \mathcal{O}_{t+1}) + (1-r_0)^2 f_{IBD}(\mathcal{I}_{t+1}),
\end{aligned}$$

Proof of b)

The proof was already given in Wellmann and Pfeiffer (2009). It is repeated here for completeness. The average kinship in the offspring is

$$
\begin{aligned}
f_{IBD}(\mathcal{O}_{t+1}) &= P(X_i = Y_j | i,j \in \mathcal{O}_{t+1}) \\
&= \frac{1}{N_0^2}\left(\sum_{\substack{i,j\in\mathcal{O}_{t+1}\\ i\neq j}} f_{ij} + \sum_{i\in\mathcal{O}_{t+1}} f_{ii}\right) \\
&= \frac{1}{N_0^2}\left(\sum_{\substack{i,j\in\mathcal{O}_{t+1}\\ i\neq j}} \frac{1}{4}(f_{s_is_j} + f_{s_id_j} + f_{d_is_j} + f_{d_id_j}) + \sum_{i\in\mathcal{O}_{t+1}} \frac{1+f_{s_id_i}}{2}\right) \\
&= \frac{1}{4N_0^2}\sum_{i,j\in\mathcal{O}_{t+1}} f_{s_is_j} + f_{s_id_j} + f_{d_is_j} + f_{d_id_j} \\
&- \frac{1}{4N_0^2}\sum_{i\in\mathcal{O}_{t+1}} (f_{s_is_i} + f_{s_id_i} + f_{d_is_i} + f_{d_id_i}) + \frac{1}{4N_0^2}\sum_{i\in\mathcal{O}_{t+1}} (2 + 2f_{s_id_i}) \\
&= \frac{1}{4N_0^2}\sum_{i,j\in\mathcal{O}_{t+1}} (f_{s_is_j} + f_{s_id_j} + f_{d_is_j} + f_{d_id_j}) + \frac{1}{4N_0^2}\sum_{i\in\mathcal{O}_{t+1}} (2 - f_{s_is_i} - f_{d_id_i}) \\
&= \frac{1}{4N_0^2}\sum_{i,j\in\mathcal{O}_{t+1}} (f_{s_is_j} + f_{s_id_j} + f_{d_is_j} + f_{d_id_j}) + \frac{1}{2N_0} - \frac{1}{4N_0^2}\sum_{i\in\mathcal{O}_{t+1}} (f_{s_is_i} + f_{d_id_i}) \\
&= \frac{1}{4N_0^2}\sum_{i\in\mathcal{O}_{t+1}}\sum_{j\in\mathcal{O}_{t+1}}\sum_{k\in\{s_i,d_i\}}\sum_{l\in\{s_j,d_j\}} f_{kl} + \frac{1}{2N_0} - \frac{1}{4N_0^2}\sum_{i\in\mathcal{O}_{t+1}}\sum_{k\in\{s_i,d_i\}} f_{kk} \\
&= \frac{1}{4N_0^2}\sum_{i\in\mathcal{O}_{t+1}}\sum_{j\in\mathcal{O}_{t+1}}\sum_{k\in\mathcal{P}_t}\sum_{l\in\mathcal{P}_t} 1_{k\in\{s_i,d_i\}}1_{l\in\{s_j,d_j\}}f_{kl} + \frac{1}{2N_0} - \frac{1}{4N_0^2}\sum_{i\in\mathcal{O}_{t+1}}\sum_{k\in\mathcal{P}_t} 1_{k\in\{s_i,d_i\}}f_{kk} \\
&= \frac{1}{4N_0^2}\sum_{k\in\mathcal{P}_t}\sum_{l\in\mathcal{P}_t} f_{kl}\left(\sum_{i\in\mathcal{O}_{t+1}} 1_{k\in\{s_i,d_i\}}\right)\left(\sum_{j\in\mathcal{O}_{t+1}} 1_{l\in\{s_j,d_j\}}\right) \\
&+ \frac{1}{2N_0} - \frac{1}{4N_0^2}\sum_{k\in\mathcal{P}_t} f_{kk}\left(\sum_{i\in\mathcal{O}_{t+1}} 1_{k\in\{s_i,d_i\}}\right) \\
&= \frac{1}{4N_0^2}\sum_{k\in\mathcal{P}_t}\sum_{l\in\mathcal{P}_t} f_{kl}\tilde{n}_k\tilde{n}_l + \frac{1}{2N_0} - \frac{1}{4N_0^2}\sum_{k\in\mathcal{P}_t} f_{kk}\tilde{n}_k \\
&= \sum_{k\in\mathcal{P}_t}\sum_{l\in\mathcal{P}_t} f_{kl}c_kc_l + \frac{1}{2N_0} - \frac{1}{2N_0}\sum_{k\in\mathcal{P}_t} f_{kk}c_k \\
&= \mathbf{c}^T\mathbf{f}_t\mathbf{c} + \frac{1 - \mathbf{c}^T d(\mathbf{f}_t)}{2N_0}.
\end{aligned}
$$

where $\tilde{n}_k = \sum_{i\in\mathcal{O}_{t+1}} 1_{k\in\{s_i,d_i\}}$ is the number of offspring of individual k, and its contribution to the next generation is $c_k = \frac{\tilde{n}_k}{2N_0}$.

Proof of c)

The average kinship of individuals born at time t or earlier that are still in the population at time $t+1$ is

$$\begin{aligned}
P(X_i = Y_j | i, j \in \mathcal{I}_{t+1}) &= \frac{1}{(1-r_0)^2} P(X_i = Y_j, i \in \mathcal{I}_{t+1}, j \in \mathcal{I}_{t+1}) \\
&= \frac{1}{(1-r_0)^2} \sum_{s_1=1}^{q} \sum_{s_2=1}^{q} P(X_i = Y_j, i \in \mathcal{P}_{s_1}^{t+1}, j \in \mathcal{P}_{s_2}^{t+1}) \\
&= \frac{1}{(1-r_0)^2} \sum_{s_1=1}^{q} \sum_{s_2=1}^{q} P(i \in \mathcal{P}_{s_1}^{t+1}) P(j \in \mathcal{P}_{s_2}^{t+1}) P(X_i = Y_j | i \in \mathcal{P}_{s_1}^{t+1}, j \in \mathcal{P}_{s_2}^{t+1}) \\
&= \frac{1}{(1-r_0)^2} \sum_{s_1=1}^{q} \sum_{s_2=1}^{q} r_{s_1}^{t+1} r_{s_2}^{t+1} P(X_i = Y_j | i \in \mathcal{P}_{s_1}^{t+1}, j \in \mathcal{P}_{s_2}^{t+1}) \\
&= \frac{\mathbf{r}_{t+1}^T \overline{\mathbf{f}}_{t+1} \mathbf{r}_{t+1}}{(1-r_0)^2}
\end{aligned}$$

where $\overline{\mathbf{f}}_{t+1}$ is a $q \times q$-matrix containing the average kinships within and between classes in the population at time $t+1$, i.e.

$$\overline{f}_{t+1, s_1 s_2} = P(X_i = Y_j | i \in \mathcal{P}_{s_1}^{t+1}, j \in \mathcal{P}_{s_2}^{t+1})$$

Unfortunately, it is unknown which individuals will contribute to the population at time $t+1$ and which individuals will be culled. Thus, matrix $\overline{\mathbf{f}}_{t+1}$ is unkown. By assuming that the individuals to be culled are randomly chosen, the matrix becomes random and the expectation can be calculated.

Since for each class $s = 1, ..., q$ the individuals in the population at time $t+1$ are a random sample of the individuals that were in the population at time t, the off-diagonal elements of matrices $E(\overline{\mathbf{f}}_{t+1})$ and $\overline{\mathbf{f}}_t$ are equal. However, since the total number of individuals in each class is reduced, the probability increases that two alleles are chosen from the same individual. Thus, the diagonal elements of matrix $E(\overline{\mathbf{f}}_{t+1})$ are slightly larger than the diagonal elements of matrix $\overline{\mathbf{f}}_t$. More precisely,

$$
\begin{aligned}
E(\overline{\mathbf{f}}_{t+1})_{ss} &= P(X_i = Y_j | i \in \mathcal{P}_s^{t+1}, j \in \mathcal{P}_s^{t+1}) \\
&= P(i \neq j | i \in \mathcal{P}_s^{t+1}, j \in \mathcal{P}_s^{t+1}) P(X_i = Y_j | i \neq j, i \in \mathcal{P}_s^{t+1}, j \in \mathcal{P}_s^{t+1}) \\
&+ P(i = j | i \in \mathcal{P}_s^{t+1}, j \in \mathcal{P}_s^{t+1}) P(X_i = Y_j | i = j, i \in \mathcal{P}_s^{t+1}, j \in \mathcal{P}_s^{t+1}) \\
&= \left(1 - \frac{1}{N_s^{t+1}}\right) P(X_i = Y_j | i \neq j, i \in \mathcal{P}_s^{t+1}, j \in \mathcal{P}_s^{t+1}) \\
&+ \frac{1}{N_s^{t+1}} P(X_i = Y_i | i \in \mathcal{P}_s^{t+1}) \\
&= \left(1 - \frac{1}{N_s^{t+1}}\right) P(X_i = Y_j | i \neq j, i \in \mathcal{P}_s^{t}, j \in \mathcal{P}_s^{t}) + \frac{1}{N_s^{t+1}} P(X_i = Y_i | i \in \mathcal{P}_s^{t}) \\
&= \left(1 - \frac{1}{N_s^{t+1}}\right) \tilde{f}_{t\,ss} + \frac{1}{N_s^{t+1}} \tilde{F}_{t,s} \\
&= \tilde{f}_{t\,ss} + \frac{1}{N_s^{t+1}} (\tilde{F}_{t,s} - \tilde{f}_{t\,ss}).
\end{aligned}
$$

Similarly,

$$
\overline{f}_{t,ss} = \tilde{f}_{t\,ss} + \frac{1}{N_s^{t}} (\tilde{F}_{t,s} - \tilde{f}_{t\,ss}).
$$

Thus, $E(\overline{\mathbf{f}}_{t+1}) = \overline{\mathbf{f}}_t + \Delta_1 \overline{\mathbf{f}}_t$ and

$$
f_{IBD}(\mathcal{I}_{t+1}) = \frac{\mathbf{r}_{t+1}^T E(\overline{\mathbf{f}}_{t+1}) \mathbf{r}_{t+1}}{(1 - r_0)^2} = \frac{\mathbf{r}_{t+1}^T \left(\overline{\mathbf{f}}_t + \Delta_1 \overline{\mathbf{f}}_t\right) \mathbf{r}_{t+1}}{(1 - r_0)^2},
$$

Proof of d)

The average kinship between the offspring and the individuals that were already born at time t, but are still in the population at time $t+1$, is

$$
\begin{aligned}
P(X_i = Y_j | i \in \mathcal{O}_{t+1}, j \in \mathcal{I}_{t+1}) &= \frac{P(X_i = Y_j, i \in \mathcal{O}_{t+1}, j \in \mathcal{I}_{t+1})}{r_0(1 - r_0)} \\
&= \frac{1}{r_0(1 - r_0)} \sum_{s=1}^{q} P(X_i = Y_j, i \in \mathcal{O}_{t+1}, j \in \mathcal{P}_s^{t+1}) \\
&= \frac{1}{r_0(1 - r_0)} \sum_{s=1}^{q} r_0 r_s^{t+1} P(X_i = Y_j | i \in \mathcal{O}_{t+1}, j \in \mathcal{P}_s^{t+1}) \\
&= \frac{1}{1 - r_0} \sum_{s=1}^{q} r_s^{t+1} P(X_i = Y_j | i \in \mathcal{O}_{t+1}, j \in \mathcal{P}_s^{t+1}),
\end{aligned}
$$

where

$$\begin{aligned}
P(X_i = Y_j | i \in \mathcal{O}_{t+1}, j \in \mathcal{P}_s^{t+1}) &= \frac{1}{N_0 N_s^{t+1}} \sum_{j \in \mathcal{P}_s^{t+1}} \sum_{i \in \mathcal{O}_{t+1}} f_{ij} \\
&= \frac{1}{N_0 N_s^{t+1}} \sum_{j \in \mathcal{P}_s^{t+1}} \sum_{i \in \mathcal{O}_{t+1}} \frac{f_{s_i j} + f_{d_i j}}{2} \\
&= \frac{1}{N_0 N_s^{t+1}} \sum_{j \in \mathcal{P}_s^{t+1}} \frac{1}{2} \sum_{i \in \mathcal{O}_{t+1}} \sum_{k \in \{s_i, d_i\}} f_{kj} \\
&= \frac{1}{N_0 N_s^{t+1}} \sum_{j \in \mathcal{P}_s^{t+1}} \frac{1}{2} \sum_{i \in \mathcal{O}_{t+1}} \sum_{k \in \mathcal{P}_t} 1_{k \in \{s_i, d_i\}} f_{kj} \\
&= \frac{1}{N_0 N_s^{t+1}} \sum_{j \in \mathcal{P}_s^{t+1}} \sum_{k \in \mathcal{P}_t} \frac{f_{kj}}{2} \sum_{i \in \mathcal{O}_{t+1}} 1_{k \in \{s_i, d_i\}} \\
&= \frac{1}{N_0 N_s^{t+1}} \sum_{j \in \mathcal{P}_s^{t+1}} \sum_{k \in \mathcal{P}_t} \frac{f_{kj}}{2} \tilde{n}_k \\
&= \frac{1}{N_s^{t+1}} \sum_{j \in \mathcal{P}_s^{t+1}} \sum_{k \in \mathcal{P}_t} c_k f_{kj} \\
&= \sum_{k \in \mathcal{P}_t} c_k \left(\frac{1}{N_s^{t+1}} \sum_{j \in \mathcal{P}_s^{t+1}} f_{kj} \right) \\
&= \sum_{k \in \mathcal{P}_t} c_k \overline{f}_{sk}^{t+1} \\
&= \mathbf{c}^T \overline{\mathbf{f}}_s^{t+1}
\end{aligned}$$

where $\overline{f}_{sk}^{t+1} = \frac{1}{N_s} \sum_{j \in \mathcal{P}_s^{t+1}} f_{kj}$ is the average kinship of individual $k \in \mathcal{P}_t$ with the individuals being in class s at time $t+1$. Thus,

$$\begin{aligned}
f_{IBD}(\mathcal{I}_{t+1}, \mathcal{O}_{t+1}) &= \frac{1}{1 - r_0} \sum_{s=1}^{q} r_s^{t+1} \mathbf{c}^T E(\overline{\mathbf{f}}_s^{t+1}) \\
&= \frac{1}{1 - r_0} \mathbf{c}^T E(\overline{\mathbf{f}}_{N \times q}^{t+1}) \mathbf{r}_{t+1} \\
&= \frac{1}{1 - r_0} \mathbf{c}^T \overline{\mathbf{f}}_{N \times q}^{t} \mathbf{r}_{t+1},
\end{aligned}$$

where $\overline{\mathbf{f}}_{N \times q}^{t+1} = (\overline{\mathbf{f}}_1^{t+1}, ..., \overline{\mathbf{f}}_q^{t+1})$, and the last equality holds because $E(\overline{\mathbf{f}}_{N \times q}^{t+1}) = \overline{\mathbf{f}}_{N \times q}^{t}$.

□

3.3 Estimate for time $t+1$

Often, the mean kinship of the population at time $t+1$ has to be predicted from a sample of individuals consisting of n_s individuals from class $s = 1, ..., q$. An (almost) unbiased estimate for the mean kinship at time $t+1$ is:

$$\hat{f}_{IBD}(\mathcal{P}_{t+1}) \quad = \quad (r_0\tilde{\mathbf{c}} + \tilde{\mathbf{v}})^T \hat{\mathbf{f}}_t (r_0\tilde{\mathbf{c}} + \tilde{\mathbf{v}}) + l_{IBD}(\tilde{\mathbf{c}}) \qquad (3)$$

where $\tilde{\mathbf{c}}$ is the vector with contributions of the individuals to the offspring. All individuals not included in the sample are assumed to have no offspring. Vector $\tilde{\mathbf{v}}$ with $\tilde{v}_i = \frac{r_s^{t+1}}{n_s}$ for $i \in \mathcal{P}_s^t$ is the proportion of the population represented by individual i. Matrix $\hat{\mathbf{f}}_t$ contains estimated pairwise kinships of the individuals in the sample. The right summand can be written as

$$\begin{aligned}
l_{IBD}(\tilde{\mathbf{c}}) &= \Delta_{00}(\tilde{\mathbf{c}}) + \Delta_{01}(\tilde{\mathbf{c}}) + \Delta_{11} \text{ with} \\
\Delta_{00}(\tilde{\mathbf{c}}) &= r_0^2 \frac{1 - \tilde{\mathbf{c}}^T d(\hat{\mathbf{f}}_t)}{2N_0}, \\
\Delta_{01}(\tilde{\mathbf{c}}) &\approx -2r_0\tilde{\mathbf{c}}^T \mathbf{u}, \\
\Delta_{11} &= \mathbf{r}_{t+1}^T \left(\Delta_1 \overline{\mathbf{f}}_t + \Delta_2 \overline{\mathbf{f}}_t\right) \mathbf{r}_{t+1},
\end{aligned}$$

where

$$u_k \quad = \quad r_{s(k)}^{t+1} \left(\frac{1}{n_{s(k)}} - \frac{1}{N_{s(k)}^t} \right) \left(f_{kk} - \tilde{f}_k \right),$$

and $\tilde{f}_k$ is the average kinship of individual k with the age cohort $s(k)$ to which it belongs. It is computed by excluding the individual itself. Moreover,

$$\Delta_2 \overline{f}_{t,s_1 s_2} \quad = \quad \left(\frac{N_{s_1 s_2}^t}{N_{s_1}^t N_{s_2}^t} - \frac{n_{s_1 s_2}}{n_{s_1} n_{s_2}} \right) \left(\tilde{F}_{t,s_1 s_2} - \tilde{f}_{t,s_1 s_2} \right),$$

where in the off-diagonals of matrix $\Delta_2 \overline{\mathbf{f}}_t$, the value $n_{s_1 s_2}$ is the number of parent-offspring pairs in the sample, and $\tilde{F}_{t,s_1 s_2}$ is the average kinship between

parents and offspring for classes s_1 and s_2. For $s_1 = s_2$, the value $n_{s_1s_2} = n_{s_1}$ is the number of individuals in class s_1, and $\tilde{F}_{t,s_1s_2}$ is the average self-kinship of individuals from class s_1.

The linear term $l_{IBD}(\tilde{\mathbf{c}})$ in Equation 3 corrects for the bias that would be obtained by using only the left summand. In particular, Δ_{00} accounts for the effect of random genetic drift on the mean kinship, Δ_{11} accounts for the fact that the sample and the population may contain different proportions of parent-offspring pairs and different numbers of individuals in each age class, and the term Δ_{01} avoids that selection candidates from age cohorts with small sample size become unduly penalized. That is, a parent k of some individuals that will be born in year $t+1$ may belong to a small sample from an age cohort of size $n_{s(k)} < N_{s(k)}^t$. Thus, without the correction term Δ_{01}, the effect of the parent on the mean kinship at time $t+1$ would be overestimated, and the use of this individual as a parent would be unduly penalised.

Proof:

The mean kinship at time $t+1$ is usually estimated from a sample. We assume that all individuals with offspring in year $t+1$ are included in the sample. An estimate of the mean kinship at time $t+1$ is

$$\tilde{f}_{IBD}(\mathcal{P}_{t+1}) \quad = \quad (r_0\tilde{\mathbf{c}} + \tilde{\mathbf{v}})^T \hat{\mathbf{f}}_t (r_0\tilde{\mathbf{c}} + \tilde{\mathbf{v}}),$$

where $\hat{\mathbf{f}}_t$ is a random submatrix of matrix $\mathbf{f}_t$, $\tilde{\mathbf{c}}$ is the corresponding subvector of vector $\mathbf{c}$, and vector $\tilde{\mathbf{v}}$ contains the contribution each individual itself has to the population at time $t+1$. It is computed as $\tilde{v}_k = \frac{r_s^{t+1}}{n_s}$, where n_s is the number of individuals in the sample from class s. However, the estimate proposed above is biased, and the term

$$l_{IBD}(\tilde{\mathbf{c}}) \quad = \quad f_{IBD}(\mathcal{P}_{t+1}) - \tilde{f}_{IBD}(\mathcal{P}_{t+1}),$$

which corrects for this bias, is derived below. We have

$$\begin{aligned}
\tilde{f}_{IBD}(\mathcal{P}_{t+1}) &= (r_0\tilde{\mathbf{c}} + \tilde{\mathbf{v}})^T \hat{\mathbf{f}}_t (r_0\tilde{\mathbf{c}} + \tilde{\mathbf{v}}) \\
&= r_0^2 \tilde{\mathbf{c}}^T \hat{\mathbf{f}}_t \tilde{\mathbf{c}} + 2r_0 \tilde{\mathbf{c}}^T \hat{\mathbf{f}}_t \tilde{\mathbf{v}} + \tilde{\mathbf{v}}^T \hat{\mathbf{f}}_t \tilde{\mathbf{v}} \\
&= r_0^2 \tilde{\mathbf{c}}^T \hat{\mathbf{f}}_t \tilde{\mathbf{c}} + 2r_0(1-r_0)\frac{\tilde{\mathbf{c}}^T \hat{\mathbf{f}}_t \tilde{\mathbf{v}}}{1-r_0} + (1-r_0)^2 \frac{\tilde{\mathbf{v}}^T \hat{\mathbf{f}}_t \tilde{\mathbf{v}}}{(1-r_0)^2} \\
&= r_0^2 \tilde{f}_{IBD}(\mathcal{O}_{t+1}) + 2r_0(1-r_0)\tilde{f}_{IBD}(\mathcal{O}_{t+1}, \mathcal{I}_{t+1}) + (1-r_0)^2 \tilde{f}_{IBD}(\mathcal{I}_{t+1}),
\end{aligned}$$

where

$$\tilde{f}_{IBD}(\mathcal{O}_{t+1}) \quad = \quad \tilde{\mathbf{c}}^T \hat{\mathbf{f}}_t \tilde{\mathbf{c}}$$

is an estimate of the mean kinship in the offspring,

$$\tilde{f}_{IBD}(\mathcal{O}_{t+1}, \mathcal{I}_{t+1}) \quad = \quad \frac{\tilde{\mathbf{c}}^T \hat{\mathbf{f}}_t \tilde{\mathbf{v}}}{1 - r_0}$$

is an estimate of the mean kinship between the offspring and the individuals that were already born at time t, and

$$\tilde{f}_{IBD}(\mathcal{I}_{t+1}) \quad = \quad \frac{\tilde{\mathbf{v}}^T \hat{\mathbf{f}}_t \tilde{\mathbf{v}}}{(1 - r_0)^2}$$

is an estimate of the mean kinship of the individuals that were already born at time t. From the corresponding equation

$$f_{IBD}(\mathcal{P}_{t+1}) \quad = \quad r_0^2 f_{IBD}(\mathcal{O}_{t+1}) + 2r_0(1 - r_0) f_{IBD}(\mathcal{O}_{t+1}, \mathcal{I}_{t+1}) + (1 - r_0)^2 f_{IBD}(\mathcal{I}_{t+1}),$$

which was derived in the previous section, we obtain

$$f_{IBD}(\mathcal{P}_{t+1}) \quad = \quad (r_0 \tilde{\mathbf{c}} + \tilde{\mathbf{v}})^T \hat{\mathbf{f}}_t (r_0 \tilde{\mathbf{c}} + \tilde{\mathbf{v}}) + l_{IBD}(\tilde{\mathbf{c}})$$

with

$$\begin{aligned}
l_{IBD}(\tilde{\mathbf{c}}) &= \Delta_{00} + \Delta_{01} + \Delta_{11}, \text{ where} \\
\Delta_{00} &= r_0^2 \left(f_{IBD}(\mathcal{O}_{t+1}) - \tilde{f}_{IBD}(\mathcal{O}_{t+1}) \right) \\
\Delta_{01} &= 2r_0(1 - r_0) \left(f_{IBD}(\mathcal{O}_{t+1}, \mathcal{I}_{t+1}) - \tilde{f}_{IBD}(\mathcal{O}_{t+1}, \mathcal{I}_{t+1}) \right) \\
\Delta_{11} &= (1 - r_0)^2 \left(f_{IBD}(\mathcal{I}_{t+1}) - \tilde{f}_{IBD}(\mathcal{I}_{t+1}) \right).
\end{aligned}$$

Since the individuals with offspring are included in the sample, we have $\tilde{\mathbf{c}}^T \hat{\mathbf{f}}_t \tilde{\mathbf{c}} = \mathbf{c}^T \mathbf{f}_t \mathbf{c}$, so

$$\begin{aligned}\Delta_{00} &= r_0^2\left(f_{IBD}(\mathcal{O}_{t+1}) - \tilde{f}_{IBD}(\mathcal{O}_{t+1})\right)\\ &= r_0^2\left(\mathbf{c}^T\mathbf{f}_t\mathbf{c} + \frac{1-\mathbf{c}^T d(\mathbf{f}_t)}{2N_0} - \tilde{\mathbf{c}}^T\hat{\mathbf{f}}_t\tilde{\mathbf{c}}\right)\\ &= r_0^2\frac{1-\mathbf{c}^T d(\mathbf{f}_t)}{2N_0}\\ &= r_0^2\frac{1-\tilde{\mathbf{c}}^T d(\hat{\mathbf{f}}_t)}{2N_0}.\end{aligned}$$

Moreover,

$$\begin{aligned}\Delta_{01} &= 2r_0(1-r_0)\left(f_{IBD}(\mathcal{O}_{t+1},\mathcal{I}_{t+1}) - \tilde{f}_{IBD}(\mathcal{O}_{t+1},\mathcal{I}_{t+1})\right)\\ &= 2r_0(1-r_0)\left(\frac{\mathbf{c}^T\overline{\mathbf{f}}^t_{N\times q}\mathbf{r}_{t+1}}{1-r_0} - \frac{\tilde{\mathbf{c}}^T\hat{\mathbf{f}}_t\tilde{\mathbf{v}}}{1-r_0}\right)\\ &= 2r_0\left(\mathbf{c}^T\overline{\mathbf{f}}^t_{N\times q}\mathbf{r}_{t+1} - \tilde{\mathbf{c}}^T\hat{\mathbf{f}}_t\tilde{\mathbf{v}}\right).\end{aligned}$$

Let $\mathbf{J}$ be the $N\times q$-matrix with $J_{is} = \frac{1}{N_s^t}$ for $i \in \mathcal{P}_s^t$, and let $\tilde{\mathbf{J}}$ be the $n\times q$-matrix with $\tilde{J}_{is} = \frac{1}{n_s}$ for $i \in \mathcal{P}_s^t$. Then we have $\tilde{\mathbf{v}} = \tilde{\mathbf{J}}\mathbf{r}_{t+1}$ and $\mathbf{v} = \mathbf{J}\mathbf{r}_{t+1}$.

Let the individuals of the population be ordered, so that the individuals from the sample come first. Since all individuals with offspring are included in the sample, we have $\mathbf{c} = \binom{\tilde{\mathbf{c}}}{\mathbf{0}}$. With $\mathbf{f}_t = \binom{\hat{\mathbf{f}}_t\ \mathbf{f}_t^{01}}{\mathbf{f}_t^{10}\mathbf{f}_t^{11}}$ and $\mathbf{J} = \binom{\mathbf{J}_0}{\mathbf{J}_1}$ we have $\overline{\mathbf{f}}^t_{N\times q} = \mathbf{f}_t\mathbf{J}$, so

$$\begin{aligned}\mathbf{c}^T\overline{\mathbf{f}}^t_{N\times q} &= \mathbf{c}^T\mathbf{f}_t\mathbf{J}\\ &= (\tilde{\mathbf{c}}^T\ 0^T)\begin{pmatrix}\hat{\mathbf{f}}_t\ \mathbf{f}_t^{01}\\ \mathbf{f}_t^{10}\mathbf{f}_t^{11}\end{pmatrix}\mathbf{J}\\ &= \left(\tilde{\mathbf{c}}^T\hat{\mathbf{f}}_t + 0^T\mathbf{f}_t^{10},\ \tilde{\mathbf{c}}^T\mathbf{f}_t^{01} + 0^T\mathbf{f}_t^{11}\right)\begin{pmatrix}\mathbf{J}_0\\ \mathbf{J}_1\end{pmatrix}\\ &= \tilde{\mathbf{c}}^T\left(\hat{\mathbf{f}}_t\mathbf{J}_0 + \mathbf{f}_t^{01}\mathbf{J}_1\right).\end{aligned}$$

Since $\tilde{\mathbf{v}} = \tilde{\mathbf{J}}\mathbf{r}_{t+1}$, we obtain

$$
\begin{aligned}
\Delta_{01} &= 2r_0\left(\mathbf{c}^T\overline{\mathbf{f}}^t_{N\times q}\mathbf{r}_{t+1} - \tilde{\mathbf{c}}^T\hat{\mathbf{f}}_t\tilde{\mathbf{v}}\right) \\
&= 2r_0\left(\tilde{\mathbf{c}}^T\left(\hat{\mathbf{f}}_t\mathbf{J}_0 + \mathbf{f}^{01}_t\mathbf{J}_1\right)\mathbf{r}_{t+1} - \tilde{\mathbf{c}}^T\hat{\mathbf{f}}_t\tilde{\mathbf{J}}\mathbf{r}_{t+1}\right) \\
&= 2r_0\tilde{\mathbf{c}}^T\left(\hat{\mathbf{f}}_t\mathbf{J}_0 + \mathbf{f}^{01}_t\mathbf{J}_1 - \hat{\mathbf{f}}_t\tilde{\mathbf{J}}\right)\mathbf{r}_{t+1} \\
&= 2r_0\tilde{\mathbf{c}}^T\left(\hat{\mathbf{f}}_t(\mathbf{J}_0 - \tilde{\mathbf{J}}) + \mathbf{f}^{01}_t\mathbf{J}_1\right)\mathbf{r}_{t+1} \\
&= 2r_0\tilde{\mathbf{c}}^T\mathbf{u},
\end{aligned}
$$

where component k of vector $\mathbf{u} = (\hat{\mathbf{f}}_t(\mathbf{J}_0 - \tilde{\mathbf{J}}) + \mathbf{f}^{01}_t\mathbf{J}_1)\mathbf{r}_{t+1}$ corresponds to the individual with contribution c_k. Take S_{s_1} to be the set of individuals from class s_1 included in the sample. We can write

$$
\begin{aligned}
u_k &= \sum_{s_1=1}^{q} r^{t+1}_{s_1}\left(\left(\frac{1}{N_{s_1}} - \frac{1}{n_{s_1}}\right)\sum_{i\in S_{s_1}}\hat{f}_{ki} + \frac{1}{N_{s_1}}\sum_{j\in P^t_{s_1}\setminus S_{s_1}} f^{01}_{kj}\right) \\
&= \sum_{s_1=1}^{q} r^{t+1}_{s_1}\frac{N_{s_1}-n_{s_1}}{N_{s_1}}\left(\frac{1}{N_{s_1}-n_{s_1}}\sum_{j\in P^t_{s_1}\setminus S_{s_1}} f^{01}_{kj} - \frac{1}{n_{s_1}}\sum_{i\in S_{s_1}}\hat{f}_{ki}\right) \\
&= \sum_{s_1=1}^{q} r^{t+1}_{s_1}\frac{N_{s_1}-n_{s_1}}{N_{s_1}}\left(\overline{f}_{k,R_{s_1}} - \overline{f}_{k,S_{s_1}}\right),
\end{aligned}
$$

where

$$
\begin{aligned}
\overline{f}_{k,R_{s_1}} &= \frac{1}{N_{s_1}-n_{s_1}}\sum_{j\in P^t_{s_1}\setminus S_{s_1}} f_{kj} \\
\overline{f}_{k,S_{s_1}} &= \frac{1}{n_{s_1}}\sum_{i\in S_{s_1}} f_{ki}
\end{aligned}
$$

If individual k is in age class s_1, it is also in the sample S_{s_1}, so it's self-kinship causes it's average kinship with the sample to be larger than it's average kinship with the individuals not included in the sample. Let $\tilde{f}_k$ denote the average kinship of individual k with the age cohort to which it belongs, excluding it's self-kinship. Then we have

$$
\begin{aligned}
\overline{f}_{k,R_{s_1}} - \overline{f}_{k,S_{s_1}} &\approx \tilde{f}_k - \frac{f_{kk} + (n_{s_1}-1)\tilde{f}_k}{n_{s_1}} \\
&= -\frac{1}{n_{s_1}}(f_{kk} - \tilde{f}_k)
\end{aligned}
$$

If individual k is not in class s_1, then it's parents could be in this age class. If the parents are in the data set and the samples from their age classes are small, then the kinship between this age class and the offspring will be overestimated if the correction term Δ_{01} does not account for this. However, since individual k has offspring, its parents are in an old age class s_1 which probably contributes only little to the population at time $t+1$, so this term can be neglected. Thus, we assume

$$\overline{f}_{k,R_{s_1}} - \overline{f}_{k,S_{s_1}} \approx 0$$

Hence,

$$\begin{aligned} u_k &= \sum_{s_1=1}^{q} r_{s_1}^{t+1} \frac{N_{s_1} - n_{s_1}}{N_{s_1}} \left(\overline{f}_{k,R_{s_1}} - \overline{f}_{k,S_{s_1}} \right) \\ &= -r_{s(k)}^{t+1} \frac{N_{s(k)} - n_{s(k)}}{N_{s(k)} n_{s(k)}} \left(f_{kk} - \tilde{f}_k \right), \end{aligned}$$

where $s(k)$ is the age class of individual k. Moreover,

$$\begin{aligned} \Delta_{11} &= (1-r_0)^2 \left(f_{IBD}(\mathcal{I}_{t+1}) - \tilde{f}_{IBD}(\mathcal{I}_{t+1}) \right) \\ &= (1-r_0)^2 \left(\frac{\mathbf{r}_{t+1}^T \overline{\mathbf{f}}_t \mathbf{r}_{t+1} + \mathbf{r}_{t+1}^T \Delta \overline{\mathbf{f}}_t \mathbf{r}_{t+1}}{(1-r_0)^2} - \frac{\tilde{\mathbf{v}}^T \hat{\mathbf{f}}_t \tilde{\mathbf{v}}}{(1-r_0)^2} \right) \\ &= \mathbf{r}_{t+1}^T \overline{\mathbf{f}}_t \mathbf{r}_{t+1} + \mathbf{r}_{t+1}^T \Delta_1 \overline{\mathbf{f}}_t \mathbf{r}_{t+1} - \tilde{\mathbf{v}}^T \hat{\mathbf{f}}_t \tilde{\mathbf{v}} \\ &= \mathbf{r}_{t+1}^T \overline{\mathbf{f}}_t \mathbf{r}_{t+1} + \mathbf{r}_{t+1}^T \Delta_1 \overline{\mathbf{f}}_t \mathbf{r}_{t+1} - \mathbf{r}_{t+1}^T \tilde{\mathbf{J}}^T \hat{\mathbf{f}}_t \tilde{\mathbf{J}} \mathbf{r}_{t+1} \\ &= \mathbf{r}_{t+1}^T \left(\Delta_1 \overline{\mathbf{f}}_t + (\overline{\mathbf{f}}_t - \hat{\overline{\mathbf{f}}}_t) \right) \mathbf{r}_{t+1} \\ &= \mathbf{r}_{t+1}^T \left(\Delta_1 \overline{\mathbf{f}}_t + \Delta_2 \overline{\mathbf{f}}_t \right) \mathbf{r}_{t+1}, \end{aligned}$$

where $\hat{\overline{\mathbf{f}}}_t = \tilde{\mathbf{J}}^T \hat{\mathbf{f}}_t \tilde{\mathbf{J}}$ contains average kinships within and between age cohorts, computed from indivdiuals from the sample, and $\Delta_2 \overline{\mathbf{f}}_t = \overline{\mathbf{f}}_t - \hat{\overline{\mathbf{f}}}_t$. Matrix $\hat{\overline{\mathbf{f}}}_t$ has larger diagonal elements than matrix $\overline{\mathbf{f}}_t$ because the probability is higher that alleles are sampled from the same individual. If the data set contains most of the parents of the individuals from the data set, then the off-diagonal elements can also be larger because the proportion of parent-offspring pairs is higher in the sample than in the population. We have

$$
\begin{aligned}
\hat{\overline{f}}_{t,s_1s_2} &= P(X_i = Y_j | i \in S_{s_1}, j \in S_{s_2}) \\
&= P((i,j) \in \mathcal{R} | i \in S_{s_1}, \jmath \in S_{s_2}) P(X_i = Y_j | (i,j) \in \mathcal{R}, i \in S_{s_1}, j \in S_{s_2}) \\
&+ P((i,j) \notin \mathcal{R} | i \in S_{s_1}, \jmath \in S_{s_2}) P(X_i = Y_j | (i,j) \notin \mathcal{R}, i \in S_{s_1}, j \in S_{s_2}) \\
&= \frac{n_{s_1s_2}}{n_{s_1}n_{s_2}} P(X_i = Y_j | (i,j) \in \mathcal{R}, i \in S_{s_1}, j \in S_{s_2}) \\
&+ (1 - \frac{n_{s_1s_2}}{n_{s_1}n_{s_2}}) P(X_i = Y_j | (i,j) \notin \mathcal{R}, i \in S_{s_1}, j \in S_{s_2}) \\
&= P(X_i = Y_j | (i,j) \notin \mathcal{R}, i \in S_{s_1}, j \in S_{s_2}) \\
&+ \frac{n_{s_1s_2}}{n_{s_1}n_{s_2}} \left(P(X_i = Y_j | (i,j) \in \mathcal{R}, i \in S_{s_1}, j \in S_{s_2}) - P(X_i = Y_j | (i,j) \notin \mathcal{R}, i \in S_{s_1}, j \in S_{s_2}) \right) \\
&\approx \tilde{f}_{t,s_1s_2} + \frac{n_{s_1s_2}}{n_{s_1}n_{s_2}} \left(\tilde{F}_{t,s_1s_2} - \tilde{f}_{t,s_1s_2} \right).
\end{aligned}
$$

Similarly,

$$
\overline{f}_{t,s_1s_2} = \tilde{f}_{t,s_1s_2} + \frac{N_{s_1s_2}}{N_{s_1}N_{s_2}} \left(\tilde{F}_{t,s_1s_2} - \tilde{f}_{t,s_1s_2} \right).
$$

Thus,

$$
\begin{aligned}
\Delta_2 \overline{f}_t &= \overline{f}_{t,s_1s_2} - \hat{\overline{f}}_{t,s_1s_2} \\
&\approx \left(\frac{N_{s_1s_2}}{N_{s_1}N_{s_2}} - \frac{n_{s_1s_2}}{n_{s_1}n_{s_2}} \right) \left(\tilde{F}_{t,s_1s_2} - \tilde{f}_{t,s_1s_2} \right).
\end{aligned}
$$

□

4 Mean native kinship in the population

4.1 Expected value at time t

The mean native kinship in the population at time t is the conditional probability that two alleles X_i, Y_j sampled from randomly chosen individuals $i, j \in \mathcal{P}_t$ are IBD, given that they are native. That is,

$$P_t(X_i = Y_j | X_i, Y_j \in \mathcal{A}_N) \quad = \quad \frac{P_t(X_i = Y_j \text{ and } X_i, Y_j \in \mathcal{A}_N)}{P_t(X_i, Y_j \in \mathcal{A}_N)},$$

where $\mathcal{A}_N$ is the set of native alleles. The nominator and the denominator can be calculated as

$$P_t(X_i = Y_j \text{ and } X_i, Y_j \in \mathcal{A}_N) \quad = \quad \mathbf{r}_t^T \left(\tilde{\mathbf{f}}_{IBD\&N} + \Delta\tilde{\mathbf{f}}_{IBD\&N} \right) \mathbf{r}_t, \qquad (4)$$

$$P_t(X_i, Y_j \in \mathcal{A}_N) \quad = \quad \mathbf{r}_t^T \left(\tilde{\mathbf{f}}_N + \Delta\tilde{\mathbf{f}}_N \right) \mathbf{r}_t, \qquad (5)$$

where $q \times q$-matrices $\tilde{\mathbf{f}}_{IBD\&N}$ and $\tilde{\mathbf{f}}_N$ are obtained from $N \times N$-matrices $\mathbf{f}_{IBD\&N}$ and $\mathbf{f}_N$. Matrix $\mathbf{f}_{IBD\&N}$ contains for each pair of individuals i, j from population $\mathcal{P}_t$ the probability that two alleles randomly chosen from both individuals are IBD and native, and matrix $\mathbf{f}_N$, contains for each pair of individuals i, j the probability that two alleles randomly chosen from both individuals are native. That is,

$$f_{IBD\&N}(i,j) \quad = \quad P_t(X_i = Y_j \text{ and } X_i, Y_j \in \mathcal{A}_N),$$
$$f_N(i,j) \quad = \quad P_t(X_i, Y_j \in \mathcal{A}_N).$$

Component (s_1, s_2) of matrix $\tilde{\mathbf{f}}_{IBD\&N}$ ($\tilde{\mathbf{f}}_N$) is the average of the values from matrix $\mathbf{f}_{IBD\&N}$ ($\mathbf{f}_N$), taken over all pairs of individuals belonging to classes s_1 and s_2, but diagonal elements and parent-offspring pairs are excluded. Matrices $\Delta\tilde{\mathbf{f}}_{IBD\&N}$ and $\Delta\tilde{\mathbf{f}}_N$ correct for the bias that would arise from excluding self-kinships and parent-offspring pairs. They are defined as

$$\Delta\tilde{f}_{IBD\&N}(s_1, s_2) \quad = \quad \frac{N_{s_1,s_2}}{N_{s_1} N_{s_2}} \left(\tilde{F}_{IBD\&N}(s_1, s_2) - \tilde{f}_{IBD\&N}(s_1, s_2) \right), \text{ and}$$
$$\Delta\tilde{f}_N(s_1, s_2) \quad = \quad \frac{N_{s_1,s_2}}{N_{s_1} N_{s_2}} \left(\tilde{F}_N(s_1, s_2) - \tilde{f}_N(s_1, s_2) \right),$$

where component (s_1, s_2) of matrix $\tilde{\mathbf{F}}_{IBD\&N}$ ($\tilde{\mathbf{F}}_N$) is computed from parent-offspring pairs and from the diagonal elements of matrix $\mathbf{f}_{IBD\&N}$ ($\mathbf{f}_N$).

Proof:

The proof of of Equations 4-5 is analogous to the proof of Equation 1. Note that

$$\begin{aligned}
\tilde{f}_{IBD\&N}(s_1,s_2) &= P(X_i=Y_j \text{ and } X_i,Y_j\in\mathcal{A}_N|i\in\mathcal{P}^t_{s_1}, j\in\mathcal{P}^t_{s_2},(i,j)\notin\mathcal{R}),\\
\tilde{f}_N(s_1,s_2) &= P(X_i,Y_j\in\mathcal{A}_N|i\in\mathcal{P}^t_{s_1}, j\in\mathcal{P}^t_{s_2},(i,j)\notin\mathcal{R}),\\
\tilde{F}_{IBD\&N}(s_1,s_2) &= P(X_i=Y_j \text{ and } X_i,Y_i\in\mathcal{A}_N|i\in\mathcal{P}^t_{s_1}, j\in\mathcal{P}^t_{s_2},(i,j)\in\mathcal{R}),\\
\tilde{F}_N(s_1,s_2) &= P(X_i,Y_j\in\mathcal{A}_N|i\in\mathcal{P}^t_{s_1}, j\in\mathcal{P}^t_{s_2},(i,j)\in\mathcal{R}).
\end{aligned}$$

□

4.2 Estimate for time $t+1$

The mean native kinship in the population at time $t+1$ is the conditional probability that two alleles X_i, Y_j sampled from randomly chosen individuals $i,j\in\mathcal{P}_{t+1}$ are IBD, given that they are native. That is,

$$\begin{aligned}
P_{t+1}(X_i=Y_j|X_i,Y_j\in\mathcal{A}_N) &= \frac{P_{t+1}(X_i=Y_j \text{ and } X_i,Y_j\in\mathcal{A}_N)}{P_{t+1}(X_i,Y_j\in\mathcal{A}_N)}\\
&= \frac{(r_0\tilde{\mathbf{c}}+\tilde{\mathbf{v}})^T\mathbf{f}_{IBD\&N}(r_0\tilde{\mathbf{c}}+\tilde{\mathbf{v}})+l_{IBD\&N}(\tilde{\mathbf{c}})}{(r_0\tilde{\mathbf{c}}+\tilde{\mathbf{v}})^T\mathbf{f}_N(r_0\tilde{\mathbf{c}}+\tilde{\mathbf{v}})+l_N(\tilde{\mathbf{c}})}, \quad (6)
\end{aligned}$$

where $\tilde{\mathbf{c}}$ is the vector with contributions of the individuals to the offspring. All individuals not included in the sample are assumed to have no offspring. Vector $\tilde{\mathbf{v}}$ with $\tilde{v}_i=\frac{r_s^{t+1}}{n_s}$ for $i\in\mathcal{P}^t_s$ is the proportion of the population represented by individual i. Matrices $\mathbf{f}_{IBD\&N}$ and $\mathbf{f}_N$ contain for each pair of individuals the probability

$$\begin{aligned}
f_{IBD\&N}(i,j) &= P_t(X_i=Y_j \text{ and } X_i,Y_j\in\mathcal{A}_N)\\
f_N(i,j) &= P_t(X_i,Y_j\in\mathcal{A}_N)
\end{aligned}$$

The linear correction terms can be calculated as

$$\begin{aligned}
l_{IBD\&N}(\tilde{\mathbf{c}}) &= \Delta^{00}_{IBD\&N}(\tilde{\mathbf{c}})+\Delta^{01}_{IBD\&N}(\tilde{\mathbf{c}})+\Delta^{11}_{IBD\&N}\\
l_N(\tilde{\mathbf{c}}) &= \Delta^{00}_N(\tilde{\mathbf{c}})+\Delta^{01}_N(\tilde{\mathbf{c}})+\Delta^{11}_N,
\end{aligned}$$

where, analogous to the corresponding formula for the kinship, the terms

$$
\begin{aligned}
\Delta^{00}_{IBD\&N}(\tilde{\mathbf{c}}) &= r_0^2 \frac{\tilde{\mathbf{c}}^T(\mathbf{NC} - d(\mathbf{f}_{IBD\&N}))}{2N_0}, \\
\Delta^{00}_{N}(\tilde{\mathbf{c}}) &= r_0^2 \frac{\tilde{\mathbf{c}}^T(\mathbf{NC} - d(\mathbf{f}_{N}))}{2N_0}
\end{aligned}
$$

account for random genetic drift. In particular, if the selected parents are inbred at the native segments, then these summands vanish, so inbred individuals seem to be favoured for breeding. As this is not desirable for a breeding program, it may be advisable to omit these summands or to replace vector $\mathbf{c}$ in these summands by a constant vector with uniform contributions. The latter is done in package optiSel. The terms

$$
\begin{aligned}
\Delta^{01}_{IBD\&N}(\tilde{\mathbf{c}}) &= -2r_0\tilde{\mathbf{c}}^T\mathbf{u}_{IBD\&N}, \\
\Delta^{01}_{N}(\tilde{\mathbf{c}}) &= -2r_0\tilde{\mathbf{c}}^T\mathbf{u}_{N}
\end{aligned}
$$

with

$$
\begin{aligned}
u_{IBD\&N}(k) &= r^{t+1}_{s(k)}\left(\frac{1}{n_{s(k)}} - \frac{1}{N^t_{s(k)}}\right)\left(f_{IBD\&N}(k,k) - \tilde{f}_{IBD\&N}(k)\right), \\
u_{N}(k) &= r^{t+1}_{s(k)}\left(\frac{1}{n_{s(k)}} - \frac{1}{N^t_{s(k)}}\right)\left(f_{N}(k,k) - \tilde{f}_{N}(k)\right),
\end{aligned}
$$

avoid that the usage of selection candidates from a class with small sample size becomes unduly penalized. Here, $\tilde{f}_{IBD\&N}(k)$ ($\tilde{f}_N(k)$) is the average of matrix $f_{IBD\&N}$ (f_N), taken over row k, and all columns corresponding to individuals from the same class, which is denoted as $s(k)$. It is computed by excluding the individual itself. The terms

$$
\begin{aligned}
\Delta^{11}_{IBD\&N}(\mathbf{c}) &= \mathbf{r}^T_{t+1}\left(\Delta_1\overline{\mathbf{f}}_{IBD\&N} + \Delta_2\overline{\mathbf{f}}_{IBD\&N}\right)\mathbf{r}_{t+1}, \\
\Delta^{11}_{N}(\mathbf{c}) &= \mathbf{r}^T_{t+1}\left(\Delta_1\overline{\mathbf{f}}_{N} + \Delta_2\overline{\mathbf{f}}_{N}\right)\mathbf{r}_{t+1}
\end{aligned}
$$

account for the fact that the sample and the population contain different numbers of individuals in each class, and a different proportion of parent-offspring pairs. Here,

$$\Delta_2\overline{f}_{IBD\&N}(s_1,s_2) = \left(\frac{N^t_{s_1s_2}}{N^t_{s_1}N^t_{s_2}} - \frac{n_{s_1s_2}}{n_{s_1}n_{s_2}}\right)\left(\tilde{F}_{IBD\&N}(s_1,s_2) - \tilde{f}_{IBD\&N}(s_1,s_2)\right),$$
$$\Delta_2\overline{f}_{N}(s_1,s_2) = \left(\frac{N^t_{s_1s_2}}{N^t_{s_1}N^t_{s_2}} - \frac{n_{s_1s_2}}{n_{s_1}n_{s_2}}\right)\left(\tilde{F}_{N}(s_1,s_2) - \tilde{f}_{N}(s_1,s_2)\right),$$

where $\tilde{f}_{IBD\&N}(s_1,s_2)$ is the average of matrix $\mathbf{f}_{IBD\&N}$ for classes s_1 and s_2, computed by excluding parents/offspring pairs and the diagonal elements. $\tilde{F}_{IBD\&N}(s_1,s_2)$ is the average of matrix $\mathbf{f}_{IBD\&N}$ for classes s_1 and s_2, computed only from parent-offspring pairs and from the diagonal elements. Matrices $\tilde{\mathbf{f}}_N$ and $\tilde{\mathbf{F}}_N$ are defined analogously.

For $s_1 \neq s_2$, the value $n_{s_1s_2}$ is the number of parent-offspring pairs in the sample, and for $s_1 = s_2$, the value $n_{s_1s_2} = n_{s_1}$ is the number of individuals in class s_1.

Matrices $\Delta\overline{\mathbf{f}}_{IBD\&N}$ and $\Delta\overline{\mathbf{f}}_N$ are diagonal matrices with

$$\Delta_1\overline{f}_{IBD\&N}(s,s) = \left(\frac{1}{N^{t+1}_s} - \frac{1}{N^t_s}\right)\left(\tilde{F}_{IBD\&N}(s,s) - \tilde{f}_{IBD\&N}(s,s)\right),$$
$$\Delta_1\overline{f}_{N}(s,s) = \left(\frac{1}{N^{t+1}_s} - \frac{1}{N^t_s}\right)\left(\tilde{F}_{N}(s,s) - \tilde{f}_{N}(s,s)\right).$$

Proof:

The proofs for the numerator and denominator of Equation 6 are analogous to the proof of Equation 3, but the following difference needs to be taken into account. The self-kinship for offspring i is

$$f_{ii} = \frac{1}{2}(1 + f_{s_id_i}),$$

but

$$f_{IBD\&N}(i,i) = \frac{1}{2}\left(NC_i + f_{IBD\&N}(s_i,d_i)\right), \text{ and}$$
$$f_N(i,i) = \frac{1}{2}\left(NC_i + f_N(s_i,d_i)\right).$$

□

4.3 Pedigree-based estimate of native kinship

The pedigree-based native kinship of individuals i, j is

$$f_{IBD|N}(i,j) = \frac{f_N(i,j)}{f_{IBD\&N}(i,j)},$$

where matrix $\mathbf{f}_N$ contains for each pair of individuals i, j the probability that alleles X_i and Y_j are both native, and matrix $\mathbf{f}_{IBD\&N}$ contains for each pair of individuals the probability that alleles X_i and Y_j are IBD and native. The matrices can be computed as

$$\begin{aligned} \mathbf{f}_N &= \frac{1}{2}\left(\mathbf{NC}\,\mathbf{1}^\top + \mathbf{1}\,\mathbf{NC}^\top\right) - \frac{1}{2}\left(\mathbf{1}\,\mathbf{1}^\top - \mathbf{f}^{FM}\right), \\ \mathbf{f}_{IBD\&N} &= \mathbf{f}_N + \mathbf{f}^M - \mathbf{f}^{FM}. \end{aligned}$$

where vector $\mathbf{NC}$ contains native contributions of the individuals. The formula for computing native contributions is given in the main text. Matrix $\mathbf{f}^{FM}$ contains for each pair of individuals the probability that 2 alleles randomly chosen from the individuals are either both native or both are from migrants, whereas matrix $\mathbf{f}^M$ contains for each pair of individuals the probability that 2 alleles randomly chosen from the population are IBD or both are from migrants.

The algorithms for computing $\mathbf{f}^{FM}$ and $\mathbf{f}^M$ are almost the same as the algorithm for computing a conventional pedigree-based kinship matrix, but particular relationship matrices are assumed for the founders. In both cases, migrant founders are unrelated to native founders. For computing $\mathbf{f}^M$ it is assumed that for migrant founders the relationship matrix is a matrix of ones, whereas for the native founders it is the diagonal matrix with diagonal elements equal to 0.5. For computing $\mathbf{f}^{FM}$ it is assumed that not only for migrant founders the relationship matrix is a matrix of ones, but also for the native founders.

Proof:

The proof was already given in Wellmann et al. (2012). It is repeated here for completeness.

In general, the pedigree-based kinship at native alleles is defined as the conditional probability that two randomly chosen alleles X, Y are IBD, given that they are native. That is,

$$P(X=Y|X\in\mathcal{A}_N,Y\in\mathcal{A}_N)=\frac{P(X=Y,\ X\in\mathcal{A}_N,\ Y\in\mathcal{A}_N)}{P(X\in\mathcal{A}_N,Y\in\mathcal{A}_N)}$$

where $\mathcal{A}_N$ is the set of alleles originating from native founders, and $\mathcal{A}_M$ is the set of alleles originating from other breeds, which are also called migrant alleles. Formulas for the numerator and denominator for computing these probabilities can be derived as follows. Since

$$\begin{aligned}
P(X\in\mathcal{A}_N,\ Y\in\mathcal{A}_N)+P(X\in\mathcal{A}_N,\ Y\in\mathcal{A}_M) &= P(X\in\mathcal{A}_N),\\
P(X\in\mathcal{A}_N,\ Y\in\mathcal{A}_N)+P(X\in\mathcal{A}_M,\ Y\in\mathcal{A}_N) &= P(Y\in\mathcal{A}_N),
\end{aligned}$$

we have

$$\begin{aligned}
P(X,Y\in\mathcal{A}_N) &= P(X\in\mathcal{A}_N,\ Y\in\mathcal{A}_N)\\
&= \frac{P(X\in\mathcal{A}_N)+P(Y\in\mathcal{A}_N)}{2}\\
&- \frac{P(X\in\mathcal{A}_N,\ Y\in\mathcal{A}_M)+P(X\in\mathcal{A}_M,\ Y\in\mathcal{A}_N)}{2}\\
&= \frac{P(X\in\mathcal{A}_N)+P(Y\in\mathcal{A}_N)}{2}-\frac{1-P(X,Y\in\mathcal{A}_N,\ \text{ or }\ X,Y\in\mathcal{A}_M)}{2}\\
&= \frac{P(X\in\mathcal{A}_N)+P(Y\in\mathcal{A}_N)}{2}-\frac{1-P(X\equiv_{FM}Y)}{2}.
\end{aligned}$$

where $P(X\equiv_{FM}Y)$ is the probability that alleles X and Y are either both native or both are from migrants. Morever,

$$\begin{aligned}
& P(X=Y,X\in\mathcal{A}_N,Y\in\mathcal{A}_N)\\
=\ & P\left(X=Y \text{ and } (X\in\mathcal{A}_N \text{ or } Y\in\mathcal{A}_N)\right)\\
=\ & P\left(X=Y \text{ and } \neg\,(X,Y\in\mathcal{A}_M)\right)\\
=\ & P\left((X=Y \text{ and } \neg\,(X,Y\in\mathcal{A}_M)) \text{ or } (X,Y\in\mathcal{A}_M)\right)-P\left(X,Y\in\mathcal{A}_M\right)\\
=\ & P\left(X=Y \text{ or } (X,Y\in\mathcal{A}_M)\right)-P(X,Y\in\mathcal{A}_M)\\
=\ & P\left(X=Y \text{ or } (X,Y\in\mathcal{A}_M)\right)\\
-\ & \left(P(X,Y\in\mathcal{A}_M \text{ or } X,Y\in\mathcal{A}_N)-P(X,Y\in\mathcal{A}_N)\right)\\
=\ & P(X,Y\in\mathcal{A}_N)+P\left(X=Y \text{ or } (X,Y\in\mathcal{A}_M)\right)\\
-\ & P(X,Y\in\mathcal{A}_M \text{ or } X,Y\in\mathcal{A}_N)\\
=\ & P(X,Y\in\mathcal{A}_N)+P\left(X=Y \text{ or } (X,Y\in\mathcal{A}_M)\right)-P(X\equiv_{FM}Y)\\
=\ & P(X,Y\in\mathcal{A}_N)+P\left(X\equiv_M Y\right)-P(X\equiv_{FM}Y),
\end{aligned}$$

where $P(X \equiv_M Y)$ is the probability that alleles X and Y are IBD or both are from migrants.

Now consider the special case that allele $X = X_i$ is chosen at random from individual i, and allele $Y = Y_j$ is chosen from individual j. We have

$$\begin{aligned} f_N(i,j) &= P(X_i, Y_j \in \mathcal{A}_N) \\ &= \frac{P(X_i \in \mathcal{A}_N) + P(Y_j \in \mathcal{A}_N)}{2} - \frac{1 - P(X_i \equiv_{FM} Y_j)}{2} \\ &= \frac{\mathrm{NC}_i + \mathrm{NC}_j}{2} - \frac{1 - f^{FM}(i,j)}{2}. \end{aligned}$$

Thus,

$$\mathbf{f}_N = \frac{1}{2}\left(\mathbf{NC}\,\mathbf{1}^\top + \mathbf{1}\,\mathbf{NC}^\top\right) - \frac{1}{2}\left(\mathbf{1}\,\mathbf{1}^\top - \mathbf{f}^{FM}\right).$$

On the other hand,

$$\begin{aligned} f_{IBD\&N}(i,j) &= P(X_i = Y_j \text{ and } X_i, Y_j \in \mathcal{A}_N) \\ &= P(X_i, Y_j \in \mathcal{A}_N) + P\left(X_i \equiv_{IM} Y_j\right) - P(X_i \equiv_{NM} Y_j) \\ &= f_N(i,j) + f^M(i,j) - f^{FM}(i,j), \end{aligned}$$

Thus,

$$\mathbf{f}_{IBD\&N} = \mathbf{f}_N + \mathbf{f}^M - \mathbf{f}^{FM}.$$

□

5 Number of parent-offspring pairs in the population at time t

Let s_k denote either a class of females of age k (in which case $s_k = f_k$), or a class of males of age k (in which case $s_k = m_k$).

The expected number of parent-offspring pairs in the population at time t with offspring being in class s_{k_1} and parents being in class $\tilde{s}_{k_2}$ is

$$N_{s_{k_1}\tilde{s}_{k_2}} = \frac{N^t_{s_{k_1}}}{N^t_{f_{k_1}} + N^t_{m_{k_1}}} N_{k_1\tilde{s}_{k_2}},$$

where $N^t_{f_k}$ ($N^t_{m_k}$) is the number of females (males) of age k still contributing to the population at time t, and $N_{k_1\tilde{s}_{k_2}}$ is the number of parent-offspring pairs in the population at time t with offspring having age k_1 and parents being in class $\tilde{s}_{k_2}$. It can be computed as

$$N_{k_1\tilde{s}_{k_2}} = N^t_{k_1} \frac{N^t_{\tilde{s}_{k_2}}}{N^t_{\tilde{s}_1}} X_{k_1,\tilde{s}_{k_2}},$$

where $N^t_{k_1}$ is the number of individuals in an age cohort that still contribute to the population at age k_1, $\frac{N^t_{\tilde{s}_{k_2}}}{N^t_{\tilde{s}_1}}$ is the proportion of individuals from class $\tilde{s}_{k_2}$ that still contribute to the population at age k_2, and

$$X_{k_1,\tilde{s}_{k_2}} = P(par_i \in P^{t-k_2+1}_{\tilde{s}_{k_2}} | i \in \mathcal{P}^t_{k_1})$$

for $\tilde{s} \in \{m, f\}$ is the probability that a parent of a randomly chosen individual of age k_1 has age k_2.

Proof:

We have

$$\begin{aligned}
N_{k_1\tilde{s}_{k_2}} &= N^t_{k_1} P(par_i \in P^t_{\tilde{s}_{k_2}} | i \in P^t_{k_1}) \\
&= N^t_{k_1} P(par_i \in P^t_{\tilde{s}_{k_2}} | par_i \in P^{t-k_2+1}_{\tilde{s}_{k_2}}) P(par_i \in P^{t-k_2+1}_{\tilde{s}_{k_2}} | i \in P^t_{k_1}) \\
&= N^t_{k_1} \frac{N^t_{\tilde{s}_{k_2}}}{N^t_{\tilde{s}_1}} P(par_i \in P^{t-k_2+1}_{\tilde{s}_{k_2}} | i \in P^t_{k_1}) \\
&= N^t_{k_1} \frac{N^t_{\tilde{s}_{k_2}}}{N^t_{\tilde{s}_1}} X_{k_1,\tilde{s}_{k_2}},
\end{aligned}$$

□
